# Conserved redox‐dependent DNA binding of ROXY glutaredoxins with TGA transcription factors

**DOI:** 10.1002/pld3.30

**Published:** 2017-12-14

**Authors:** Nora Gutsche, Michael Holtmannspötter, Lucia Maß, Martin O'Donoghue, Andrea Busch, Andrea Lauri, Veit Schubert, Sabine Zachgo

**Affiliations:** ^1^ Botany Department School of Biology and Chemistry Osnabrück University Osnabrück Germany; ^2^ Teagasc Ashtown Food Research Centre Dublin Ireland; ^3^ Axxam S.p.A. Bresso‐Milan Italy; ^4^ Leibniz Institute of Plant Genetics and Crop Plant Research (IPK) Stadt Seeland Germany

**Keywords:** active/inactive RNA polymerase II, FRET‐FLIM, *Marchantia polymorpha*, Mp*ROXY1/2*, Mp*TGA*, redox‐dependent DNA binding

## Abstract

The *Arabidopsis thaliana* CC‐type glutaredoxin (GRX) ROXY1 and the bZIP TGA transcription factor (TF) PERIANTHIA (PAN) interact in the nucleus and together regulate petal development. The CC‐type GRXs exist exclusively in land plants, and in contrast to the ubiquitously occurring CPYC and CGFS GRX classes, only the CC‐type GRXs expanded strongly during land plant evolution. Phylogenetic analyses show that TGA TFs evolved before the CC‐type GRXs in charophycean algae. Mp*ROXY1/2* and Mp*TGA* were isolated from the liverwort *Marchantia polymorpha* to analyze regulatory ROXY/TGA interactions in a basal land plant. Homologous and heterologous protein interaction studies demonstrate that nuclear ROXY/TGA interactions are conserved since the occurrence of CC‐type GRXs in bryophytes and mediated by a conserved ROXY C‐terminus. Redox EMSA analyses show a redox‐sensitive binding of MpTGA to the *cis*‐regulatory *as‐1‐*like element. Furthermore, we demonstrate that MpTGA binds together with MpROXY1/2 to this motif under reducing conditions, whereas this interaction is not observed under oxidizing conditions. Remarkably, heterologous complementation studies reveal a strongly conserved land plant *ROXY* activity, suggesting an ancestral role for CC‐type GRXs in modulating the activities of TGA TFs. Super‐resolution microscopy experiments detected a strong colocalization of ROXY1 with the active form of the RNA polymerase II in the nucleus. Together, these data shed new light on the function of ROXYs and TGA TFs and the evolution of redox‐sensitive transcription regulation processes, which likely contributed to adapt land plants to novel terrestrial habitats.

## 1 INTRODUCTION

Glutaredoxins (GRXs) are small thioredoxin‐fold family proteins that can act as glutathione‐dependent oxidoreductases via an active site motif comprising at least one cysteine residue (Couturier, Przybyla‐Toscano, Roret, Didierjean, & Rouhier, 2015; Lillig & Berndt, 2013). Based on the motif sequence, GRXs can be grouped into three main classes. The CPYC (class I) and CGFS (class II) GRXs occur ubiquitously in prokaryotes and eukaryotes. They can bind Fe‐S cluster and are known to function in oxidative stress response (class I) or in iron‐sulfur cluster biogenesis (class II; Bräutigam et al., 2013; Couturier et al., 2015; Li & Outten, 2012; Moseler et al., 2015). Differently, class III CC‐type GRXs exclusively evolved in land plants, raising the intriguing question whether and how they contributed to an adaptation to the new terrestrial environment. Interestingly, whereas the number of the class I and class II GRXs remained rather similar throughout land plant evolution, only the CC‐type GRX class strongly expanded over the last 450 MY (Ziemann, Bhave, & Zachgo, 2009). The first analyzed *Arabidopsis thaliana* CC‐type loss‐of‐function mutant, named *roxy1*, revealed a crucial function of *ROXY1* during flower development (Xing, Rosso, & Zachgo, 2005). The *roxy1* mutant forms only 2.5 instead of 4.0 petals and also, later petal morphogenesis is disturbed. Analysis of its closest homolog *ROXY2* showed that these two CC‐type GRXs act together in microsporogenesis (Xing & Zachgo, 2008). Similarly, the switch from somatic to germline cell formation was also shown to be under the control of the monocot *ROXY* homologs *MICROSPORELESS1* (*MIL1*) from *Oryza sativa* and *MALE STERILE CONVERTED ANTHER1* (*MSCA1*) from *Zea mays* (Hong et al., 2012; Kelliher & Walbot, 2012).

The bZIP TGA transcription factors (TFs) were identified as ROXY1 interaction partners (Li et al., 2009; Zander, Chen, Imkampe, Thurow, & Gatz, 2012). It was shown that ROXY1 interacts in the nucleus with the class V TGA TF PERIANTHIA (PAN) and that both genes act in the same regulatory pathway (Li et al., 2009). Similarly, the maize CC‐type GRX *MSCA1* binds the TGA TF *FASCIATED EAR4* (*FEA4*) and both genes control meristem size (Pautler et al., 2015; Yang et al., 2015). The interaction of ROXYs with TGA TFs is mediated via a conserved ROXY C‐terminus, which contains an LxxL/xL and ALWL motif (Li, Gutsche, & Zachgo, 2011; Zander et al., 2012). The 10 *A. thaliana* TGA TFs group into five classes (I–V) and share the characteristic bZIP domain with a nuclear localization sequence (NLS) and two glutamine‐rich regions (Q1/Q2) in the C‐terminal part (Gatz, 2013; Jakoby et al., 2002). TGA TFs are known to bind to short *cis*‐regulatory DNA elements, such as the *as‐1*,* as‐1‐*like, and *AAGAAT* motif, all containing the name‐giving TGACG core motif (Krawczyk, Thurow, Niggeweg, & Gatz, 2002; Lebel et al., 1998; Maier et al., 2009). Redox regulation of TFs has been well documented in prokaryotes and nonplant eukaryotes and is also emerging as a concept for regulating the activity of plant TFs in stress acclimation and development (Dietz, 2014). For PAN, redox sensitivity of DNA binding has recently been shown, where binding is strongly reduced under oxidizing conditions. This sensitivity is mediated conjointly by five cysteine residues in an N‐terminal PAN extension, exclusively found in Brassicaceae homologs (Gutsche & Zachgo, 2016). For a PAN in vivo function, Cys340 is indispensable (Li et al., 2009), further supporting redox modulation of the PAN activity. Interestingly, analysis of the maize CC‐type GRX *msca1* mutant showed that hypoxia, which naturally occurs during plant growth, triggers male meiotic fate acquisition. This supports a function for MSCA1 interacting with the TGA TF FEA4 in reprogramming cell fates in response to altered redox conditions (Kelliher & Walbot, 2012). Besides these developmental ROXY/TGA functions shown by genetic studies, the combination of overexpression studies in *A. thaliana* together with in vitro and yeast interaction analyses supports the idea that ROXY18/19 and TGA2/5/6 function together in stress‐related processes, where redundant ROXY activities seem to hamper functional analyses (Herrera‐Vasquez et al., 2015; Huang et al., 2016; La Camera et al., 2011; Ndamukong et al., 2007; Zander et al., 2012).

In this study, we aimed to determine (I) the origin of the plant regulatory ROXY/TGA interaction network. We analyzed whether (II) a basal land plant TGA TF alone binds redox sensitively to *cis*‐regulatory elements and (III) also in a complex together with ROXYs. Finally, (IV) we investigated the subnuclear ROXY1 localization in correlation with active transcription processes by super‐resolution microscopy. Toward these goals, we took advantage of the liverwort *Marchantia polymorpha*. This basal land plant belongs together with mosses and hornworts to the bryophyte group, among which phylogenetic relationships are not yet unambiguously resolved (Qiu et al., 2006; Wickett et al., 2014). *M. polymorpha* contains most of the land plant gene families, however with lower gene numbers (Bowman et al., 2017; Catarino, Hetherington, Emms, Kelly, & Dolan, 2016). Differently from the moss *Physcomitrella patens*, this liverwort did not experience a paleopolyploidization event (Rensing et al., 2008). *M. polymorpha* possesses only two CC‐type GRXs, Mp*ROXY1* and Mp*ROXY2,* and one single TGA TF, Mp*TGA,* and is thus an ideal organism to analyze the origin and evolution of ROXY/TGA interactions and their regulatory nuclear activities.

## 2 MATERIALS AND METHODS

### 2.1 Sequence analyses

GRX and TGA TF sequences, listed in Table S1, were obtained from selected land plant species using pico‐PLAZA 2.0 (Vandepoele et al., 2013), Gymno PLAZA 1.0, Monocots PLAZA 3.0, Dicots PLAZA 3.0 (Van Bel et al., 2012), Congenie (Sundell et al., 2015), Phytozome v11.0 (Goodstein et al., 2012), and from transcriptome data provided by Cooper, Endymion; Delwiche, Charles (2016, figshare.https://doi.org/10.6084/m9.figshare.1604778), conducting a combination of blastp, tblastx, and blastn searches. Protein sequences were identified based on homologies and the presence of conserved domains (bZIP and Q‐rich regions for TGA TFs) or motifs (CCxx, CxxC, and CGxS motifs for GRXs) from *Ostreococcus lucimarinus, Chlamydomonas reinhardtii, Klebsormidium flaccidum, Nitella mirabilis, Coleochaete orbicularis, Spirogyra pratensis, Marchantia polymorpha*,* Physcomitrella patens*,* Selaginella moellendorffii*,* Picea abies*,* Pinus taeda*,* Brachypodium distachyon, Oryza sativa, Mimulus guttatus, Populus trichocarpa*, and *Arabidopsis thaliana*. Accession numbers for the analyzed sequences are listed in Table S1.

### 2.2 Complementation of the *A. thaliana roxy1‐2* mutant

Mp*ROXY1* (Mapoly0048s0012) and Mp*ROXY2* (Mapoly0059s0028) CDS were amplified from *M. polymorpha* thallus cDNA (*M. polymorpha*, ssp. *ruderalis*, ecotype BoGa, Osnabrück) using primers containing *Xba*I sites (MpROXY1F/R, MpROXY2F/R) and cloned downstream of a 3.6‐kb *ROXY1* promoter fragment in the pGSA1252 vector by *Xba*I restriction sites as described before (Wang, Xing, Birkenbihl, & Zachgo, 2009). All generated vectors were confirmed by sequencing and transformed into *roxy1‐2* mutants mediated by the *Agrobacterium tumefaciens* strain GV3101 pMP90 (Wang et al., 2009). Transgenic plants were selected by spraying with a 0.2% (v/v) BASTA^®^ solution. *A. thaliana* plants were grown in the glasshouse under controlled environmental conditions with 16‐hr light and 8‐hr dark. Primers from all experiments are listed in Table S2.

### 2.3 In planta interaction studies using BiFC and FRET‐FLIM

Bimolecular fluorescence complementation (BiFC) studies were performed as described by Li et al. (2009). Full‐length Mp*ROXY1/2* and Mp*TGA* sequences were inserted into Gateway^®^‐compatible YN and YC vectors, respectively, and used for Agrobacterium‐mediated transient transformation of *Nicotiana benthamiana* leaves. Images were captured with a Zeiss 510 META NLO using a Plan‐Apochromat 20x/0.8 objective. YFP was excited via a 515‐nm argon laser, and the emission was detected using a 535‐ to 590‐nm band‐pass filter.

For FRET‐FLIM analysis, 2in1 expression vectors with mTURQUOISE2 (mTRQ2) and mVENUS were generated as described in Hecker et al. (2015) using the pDONR221‐P3P2 and pDONR221‐P1P4 donor vectors and the pFRET‐TV‐NN destination vector containing the two fluorophores 5′ of Gateway^®^ cassettes. Mp*TGA* and Mp*TGA3xC*, a variant where all cysteines are substituted by serines, were N‐terminally fused with mTRQ2. Mp*ROXY1, MpROXY2,* Mp*ROXY1Δ14,* and the Mp*ROXY1* C‐terminal 14 amino acids alone (14AA) were N‐terminally fused with mVENUS. FLIM measurements were taken using an Olympus LSM FV1000 confocal microscope equipped with a FLIM unit (PicoQuant) using an UPLSAPO 60x/1.20 water‐immersion objective. FLIM data were obtained from 40 nuclei from four individual leaves of two 3‐ to 4‐week‐old *N. benthamiana* plants. mTRQ2 was excited with a 440‐nm pulsed laser diode (LDH‐P‐C‐440B) with a repetition rate of 40 MHz connected to the computer‐controlled multichannel picosecond diode laser driver Sepia II (PDL 828, PicoQuant). Emission was detected via a single‐photon avalanche detector equipped with a 465‐ to 500‐nm band‐pass filter (BrightLine HC 482/35) by time‐correlated single‐photon counting using a PicoHarp 300 module with the SymPhoTime 64 software (PicoQuant). Time‐correlated single‐photon histograms were reconvoluted with an estimated instrument response function and fitted against a monoexponential decay function for noninteracting proteins and against a biexponential decay function for interacting proteins. The average mTRQ2 lifetimes and resulting standard deviations were calculated using Microsoft excel.

### 2.4 In situ hybridization

For the detection of endogenous Mp*ROXY1/2* and Mp*TGA* mRNA, fixation and sectioning of *M. polymorpha* thallus and dig‐labeled antisense RNA preparation, hybridization and detection was carried out according to Zachgo (2002). For RNA transcription, PCR templates comprising a T7 RNA polymerase binding sites (Table S2) were generated using *M. polymorpha* cDNA, isolated as described by Busch and Zachgo (2007).

### 2.5 In vitro DNA binding assays

Redox EMSA studies were performed as described previously (Gutsche & Zachgo, 2016) using commercial *as‐1*‐like*, Δas‐1*‐like, *AAGAAT,* and *ΔbZIP* oligonucleotides 5′‐labeled with 6‐carboxyfluorescein (6‐FAM, Sigma‐Aldrich, Table S2). CDS of Mp*TGA* and the cysteine‐to‐serine mutagenized Mp*TGAC143S*, Mp*TGAC199S*, Mp*TGAC231S,* and Mp*TGA3xC* variants, produced by paired mutagenic oligomers, were cloned into the pTNT™ vector (Promega) with an N‐terminal 6xHis‐tag using *Kpn*I (5′) and *Xba*I (3′) restriction sites. Verified plasmids were used for in vitro protein expression in binding assays, as described by Gutsche and Zachgo (2016). The CDS of Mp*ROXY1*, Mp*ROXY1Δ14,* Mp*ROXY2,* and *PAN* were introduced into the pMAL‐c5X vector (NEB) using *Xmn*I (5′) and *Bam*HI (3′) sites to generate proteins fused N‐terminally with the maltose‐binding protein (MBP). Verified plasmids were transformed into the BL21 (DE3) *E. coli* strain (Novagen). Recombinant proteins were expressed and purified according to the manufacturer's protocol, and 500 ng of protein was added to each in vitro binding reaction. For the expression of ROXY proteins, 2 mM ammonium iron(III) citrate (Sigma) was supplemented to the media. Fluorophore detection was carried out with the ChemiDoc™ MP imaging system (Bio‐Rad) using the 530/28‐nm filters and blue epiillumination and the Image Lab™ software (5.0 version). All EMSA experiments were repeated at least three times using different translation products for the in vitro translated MpTGAs.

### 2.6 Overexpression studies in *M. polymorpha*

Mutagenized Mp*ROXY1‐OXAAMA* and Mp*ROXY2‐OXAAVA* CDS were produced via site‐directed mutagenesis using oligonucleotides and overlap PCRs as described by Li et al. (2009). The mutagenized sequences and the Mp*ROXY1*/2 CDS were amplified with primers containing *att*B sites allowing the recombination in the pDONR™ 207 vector using the Gateway^®^ technology (Invitrogen). Verified vectors were used in LR recombination reactions according to the manufacturer's instructions together with a modified pGWB2 vector comprising the Mp*EF1*α promoter from *M. polymorpha* (Althoff et al., 2014). To generate N‐terminal mCHERRY fusion proteins, the *mCHERRY* sequence was inserted between the Mp*EF1*α promoter and the Gateway^®^ cassette via restriction sites. Constructs were transformed into *M. polymorpha* sporelings (*M. polymorpha*, ssp. *ruderalis*, ecotype BoGa, Osnabrück) as described by Ishizaki, Chiyoda, Yamato, and Kohchi (2008) using the Agrobacterium strain C58C1 pGV2260. 100 T1 transformants were randomly selected for each construct, placed on new selection media, and phenotypically analyzed after 4 weeks of growth under 16‐hr light/8‐hr dark. Pictures of the T1 lines were captured using a Leica M165 FC stereomicroscope.

### 2.7 Preparation of *A. thaliana* root meristem nuclei and immunolocalization

The binary vector pGSA1252 containing the 3.6‐kb *ROXY1* promoter fragment and the *ROXY1* CDS, known to complement the *roxy1* flower phenotype (Li et al., 2009; Xing et al., 2005), was used to generate *pROXY1:GFP‐ROXY1* constructs. *Xba*I sites were introduced at the 5′ and 3′ ends of the *GFP* CDS, and the construct was ligated into the binary vector. Four T1 lines that complemented the *roxy1‐2* petal phenotype and revealed a strong GFP expression during early flower development were selected and the respective T2 to T4 plants were used for the immunolocalization experiments as described by Lermontova et al. (2006). Briefly, seeds were cultivated on germination media for 2 days and fixed and the cell walls digested before squashing primary roots on Polysine™ slides (Thermo Scientific). After freezing in liquid nitrogen, the coverslips were removed with a razor blade and the slides were immediately transferred into 1× MTSB (50 mM PIPES, 5 mM MgSO_4_, 5 mM EGTA, pH 6.9). The immunostaining to localize active and inactive RNA polymerase II (RNAPII) was performed according to Jasencakova, Meister, Walter, Turner, and Schubert (2000). Nonphosphorylated, transcriptionally inactive RNAPII was detected using primary mouse monoclonal antibodies (Abcam) and secondary donkey anti‐mouse‐Cy5 antibodies (Jackson ImmunoResearch). Transcriptionally active RNAPIIS2P, phosphorylated at Ser2, was detected by rat monoclonal antibodies (Millipore) and donkey anti‐rat‐Cy3 antibodies (Jackson ImmunoResearch). Polyclonal goat anti‐GFP‐DyLight488 antibodies (Rockland) were employed for direct immunostaining of GFP‐ROXY1 fusion proteins. The nuclei were counterstained with 4′,6‐diamidino‐2‐phenylindole (DAPI, 1 μg/ml) in Vectashield (Vector Laboratories). The Pearson's correlation coefficient was calculated using the image‐processing program Imaris 7.4 (Bitplane AG).

### 2.8 Super‐resolution microscopy (SIM)

For super‐resolution analyses, structured illumination microscopy (SIM) was applied using a Plan‐Apochromat 63x/1.4 oil objective of a Zeiss ELYRA PS.1 microscope and the software ZEN (Carl Zeiss GmbH). Images were captured separately for each fluorophore using appropriate excitation and emission filters. Optimal grid sizes for each wavelength were chosen according to the recommendation of the manufacturer. For 3D‐SIM, image stacks with a step size of 110 nm were acquired sequentially for each fluorophore, starting with the highest wavelength dye. In total, 22 nuclei were analyzed and processed using the software ZEN (Schubert & Weisshart, 2015). The image stacks were further analyzed for colocalization using the Imaris 7.4 (Bitplane) software. After applying automatic thresholding to exclude intensity pairs not exhibiting a correlation, the Pearson's correlation coefficient was calculated and quantitatively assessed. The whole nucleus volume from the SIM image stacks was calculated using the Imaris 7.4 tool *volume rendering*. Screenshots of the 3D reconstructed nucleus show an overview of the labeled subnuclear structures.

## 3 RESULTS

### 3.1 Evolution of CC‐type GRXs and TGA TFs in viridiplantae

**Figure 1:**
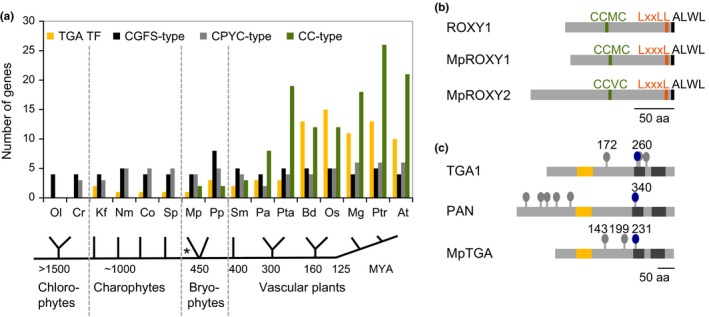
Analysis of three GRX classes and TGA transcription factors in green plants. (a) CC‐type GRXs are absent in the chlorophytes and charophytes and only exist in bryophytes and vascular plants. CC‐type GRX numbers strongly increased during land plant evolution in contrast to the ubiquitously occurring CPYC and CGFS GRXs, where family sizes remained rather similar. TGA TFs are present in charophytes and expanded in angiosperms. Approximate evolutionary times are indicated in MYA based on Ziemann et al. (2009) and Becker and Marin (2009). Ol, *Ostreococcus lucimarinus;* Cr, *Chlamydomonas reinhardtii;* Kf, *Klebsormidium flaccidum;* Nm, *Nitella mirabilis;* Co, *Coleochaete orbicularis;* Sp, *Spirogyra pratensis;* Mp, *Marchantia polymorpha;* Pp, *Physcomitrella patens*; Sm, *Selaginella moellendorffii;* Pa, *Picea abies;* Pta, *Pinus taeda*; Bd, *Brachypodium distachyon;* Os, *Oryza sativa;* Mg, *Mimulus guttatus;* Ptr, *Populus trichocarpa;* and At, *Arabidopsis thaliana*. Accession numbers of the analyzed sequences are listed in Table S1. * indicates the unresolved phylogenetic order of liverworts and mosses in the paraphyletic bryophyte group. (b) Primary structures of the CC‐type GRXs ROXY1 (136 aa, At3g02000), MpROXY1 (130 aa, Mapoly0048s0012), and MpROXY2 (179 aa, Mapoly0059s0028). Indicated are the active site motifs and the conserved C‐terminal LxxL/xL and ALWL motifs. Bar = 50 aa. (c) TGA TF comparison showing the structures of TGA1 (369 aa, At5g65210), PAN (452 aa, At1g68640), and MpTGA (341 aa, Mapoly0026s0039). The bZIP domain (yellow bars) and Q1/Q2 (dark gray bars) domains are depicted and cysteines indicated. Position equivalents of the PAN Cys340 are labeled in blue. Bar = 50 aa

Previous CPYC, CGFS, and CC‐type GRX phlyogenetic analyses (Couturier, Jacquot, & Rouhier, 2009; Ziemann et al., 2009) were extended to include updated sequence data and novel information from four charophycean algae (Figure 1a; Fig. S1 and Table S1). In contrast to the ubiquitously occurring CPYC and CGFS GRXs, CC‐type GRXs were not identified in the charophycean algae, corroborating that CC‐type GRXs exclusively evolved in land plants. Currently, the exact phylogenetic relationships of the early land plant lineages remain unresolved (Wickett et al., 2014). The existence of two CC‐type GRXs named Mp*ROXY1* and Mp*ROXY2* in the liverwort *M. polymorpha* and two orthologs in the moss *P. patens* argues that this novel GRX class likely evolved in the last common ancestor of the bryophytes (Figure 1a; Fig. S1). Interestingly, our analysis shows that the earliest emerging bryophyte CC‐type ROXYs already contain a C‐terminus with LxxL/xL and ALWL motifs (Figure 1b). This C‐terminus was shown to mediate the interaction of *A. thaliana* ROXYs with TGA TFs (Li et al., 2011; Zander et al., 2012) and its presence in MpROXY1/2 suggests that these GRXs are competent to interact with TGA TFs since they evolved. With respect to the CC‐type motif, MpROXY1 has a CCMC sequence identical to ROXY1 and the CCVC motif of MpROXY2 shows a minor deviation.

The origin of TGA TFs has thus far not been investigated, and we therefore aimed to determine the occurrence and expansion dynamics of this bZIP TF group in comparison with the GRXs. TGA TFs do not exist in chlorophytes. However, in contrast to the CC‐type GRXs, TGA TFs were identified in the charophycean algae *Klebsormidium flaccidum, Nitella mirabilis, Coleochaete orbicularis*, and *Spirogyra pratensis* (Figure 1a; Fig. S2). Therefore, TGA TFs likely evolved before the CC‐type GRXs in the last common ancestor of charophycean algae. One TGA TF, named Mp*TGA*, exists in *M. polymorpha* and groups together with TGA TFs from the charophytes, bryophytes, and lycophytes (Fig. S2). Three TGA TFs are present in *P. patens*, where the number likely increased by paleoploidization events that occurred in this moss lineage (Rensing et al., 2008). Coinciding with the strong CC‐type GRX radiation in angiosperms, TGA TFs also increased up to 11 in *Mimulus guttatus* and 13 and 10 in *Populus trichocarpa* and *A. thalian*a, respectively. MpTGA contains the bZIP domain including a NLS and the characteristic glutamine‐rich domains Q1/Q2 that may function as transcription activation domains (Gatz, 2013). In Arabidopsis, Cys340 in the PAN Q1 domain was shown to be functionally important for governing petal development (Li et al., 2009) and its Cys260 equivalent from TGA1 regulates protein activity via an intramolecular disulfide bridge formation with Cys266 (Despres et al., 2003). MpTGA possesses the three cysteine residues Cys143, Cys199, and Cys231, the latter representing the position equivalent to PAN Cys340 and TGA1 Cys260 (Figure 1c).

### 3.2 Mp*ROXY1/2* rescue petal defects in *A. thaliana roxy1‐2* mutants

**Figure 2:**
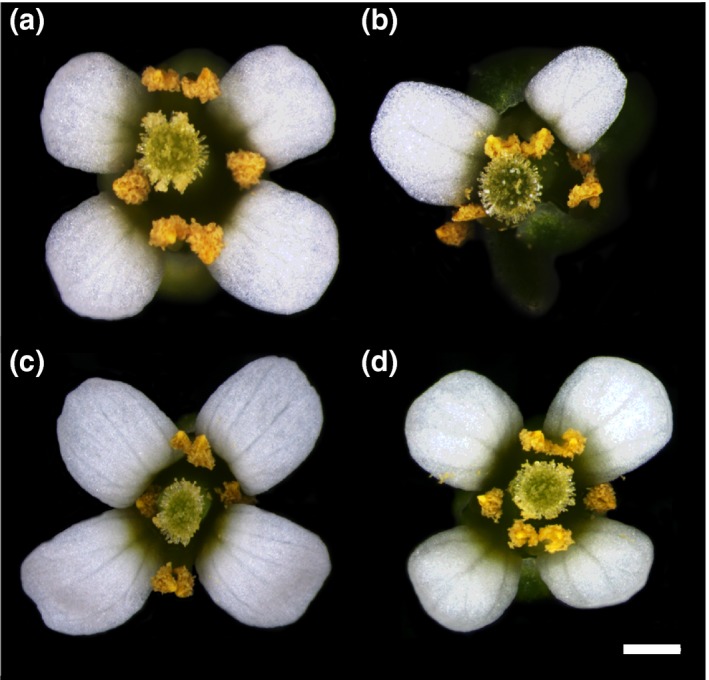
Complementation of the *roxy1‐2* mutant by Mp*ROXY1* and Mp*ROXY2*. Mp*ROXY1* and Mp*ROXY2* were expressed under the control of the endogenous *A. thaliana ROXY1* promoter and transformed into the *roxy1‐2* mutant. (a) *A. thaliana* wild‐type flowers form four equally shaped petals. (b) A typical *roxy1‐2* mutant flower produces less and abnormally formed petals. Representative flowers from T1 *A. thaliana* transgenic plants harboring the *pROXY1*:Mp*ROXY1* (c) and *pROXY1*:Mp*ROXY2* (d) constructs in a *roxy1‐2* background produce wild‐type‐like flowers with four normal petals. Bar = 500 μm

Previous research showed that several *A. thaliana ROXY* family members and even more distantly related homologs, such as Os*ROXY1* and Os*ROXY2* from rice, can functionally replace the *ROXY1* activity when they contain a conserved C‐terminus encompassing the LxxL/xL and ALWL motifs (Li et al., 2009; Wang et al., 2009). Here, we tested whether the *M. polymorpha* orthologs Mp*ROXY1/2* can govern *A. thaliana* flower development. Complementation vectors were constructed by expressing the coding regions of Mp*ROXY1* and Mp*ROXY2* under the control of the 3.6‐kb *ROXY1* promoter sequences, which has been shown to confer an endogenous *ROXY1* expression (Xing et al., 2005). The Mp*ROXY1/2* constructs were transformed along with an empty vector control into *roxy1‐2* mutants and flower phenotypes of 60 transgenic T1 plants were analyzed for each construct. Control *roxy1‐2* plants formed the typical *roxy1* mutant petal phenotype and developed on average instead of 4.0 only 2.5 petals (Figure 2a,b). 58 of 60 T1 *roxy1‐2 pROXY1*:Mp*ROXY1* plants formed wild‐type‐like flowers with four petals (Figure 2c). Similarly, although with a slightly reduced efficiency, 43 of 60 T1 *roxy1‐2 pROXY1*:Mp*ROXY2* plants developed normal petals (Figure 2d). Mp*ROXY1* and Mp*ROXY2* can thus exert the same function as *ROXY1* in *A. thaliana* petal development, revealing that their activity has been highly conserved since the emergence of CC‐type GRXs in a common bryophyte ancestor.

### 3.3 MpROXY1/2 and TGA TF proteins interact in planta

Yeast two‐hybrid analyses were conducted to test whether MpROXYs can interact with MpTGA and PAN (Fig. S3). The interaction strength for tested protein pairs using MpROXY1/2 as prey (AD‐MpROXY1/2) and MpTGA/PAN as bait (BD‐MpTGA/PAN) was quantified by determining ß‐galactosidase reporter gene expression. MpROXY1 interacts with MpTGA and also with PAN and weaker interactions were detected for MpROXY2 with MpTGA and PAN. The removal of the C‐terminal 14 amino acids of MpROXY1 containing the LxxL/xL and ALWL motifs precluded an interaction with MpTGA.

**Figure 3:**
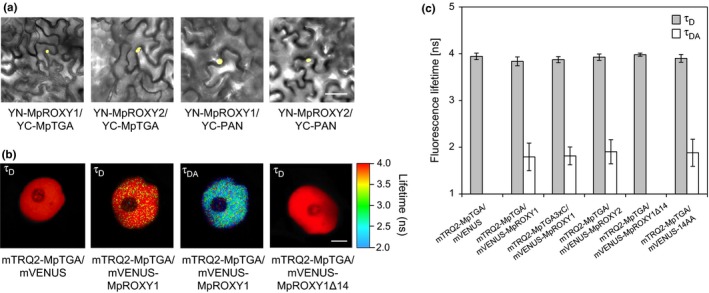
In planta MpROXY1/2 and MpTGA protein interactions. (a) BiFC interaction analyses show an interaction of MpROXY1/2 with MpTGA and PAN in nuclei of *N. benthamiana* epidermal leaf cells. (b) Representative FLIM images of *N. benthamiana* nuclei (*n* = 40) expressing mTRQ2‐MpTGA together with mVENUS alone, mVENUS‐MpROXY1, and mVENUS‐MpROXY1Δ14 fusion proteins, the latter was lacking the C‐terminal 14 amino acids containing the LxxL/xL and ALWL motifs. τ_D_, donor‐only fluorescence lifetime; τ_DA_, donor fluorescence lifetime in the presence of acceptor molecules. (c) τ_D_ and τ_DA_ for mTRQ2‐MpTGA/MpTGA3xC and mVENUS‐MpROXY1/2 interactions (*n* = 40). For mVENUS‐14AA, only the 14 C‐terminal MpROXY1 amino acids were fused with mVENUS. No second, shortened lifetime (τ_DA_) could be measured indicating the absence of an interaction, which was also observed for the control mTRQ2‐MpTGA/mVENUS. Scale bars (a) =50 μm, (b) =2 μm. (c) Error bars indicate *SD*

Next, bimolecular fluorescence complementation (BiFC) experiments were performed to analyze the interactions in planta. MpROXY1 and MpROXY2 were N‐terminally fused with the N‐terminus of the yellow fluorescent protein (YN‐MpROXY1, YN‐MpROXY2), while the C‐terminus of YFP was N‐terminally fused to MpTGA (YC‐MpTGA) and PAN (YC‐PAN). Four days after coinfiltration of the constructs into tobacco leaf epidermis cells, reconstitution of a nuclear YFP fluorescence was observed for the analyzed homologous and also heterologous protein interactions, whereas no fluorescence was detected in control experiments (Figure 3a; Fig. S4A). As described previously for PAN and ROXY1 (Li et al., 2009), analysis of GFP‐MpTGA and GFP‐MpROXY1/2 fluorescence revealed a nuclear GFP‐MpTGA localization. A cytosolic and nuclear localization was detected for GFP‐MpROXY1/2, resembling the control where GFP protein was expressed alone (Fig. S4B). The cross‐complementation data showed the capacity of the *M. polymorpha* ROXYs to replace the ROXY1 function and the heterologous Y2H and BiFC data support that this is likely mediated by conserved interactions between ROXYs and TGA TFs.

A quantitative interpretation of BiFC data is difficult due to possible variations in protein expression levels. We applied the 2in1 cloning strategy to couple in planta Förster resonance energy transfer (FRET) studies with fluorescence lifetime microscopy (FLIM) analyses (FRET‐FLIM; Hecker et al., 2015), which also allowed to determine the impact of the MpROXY1 C‐terminus and the three MpTGA cysteines on the interaction capacity. The acceptor fluorophore mVENUS (Nagai et al., 2002) was fused N‐terminally to MpROXY1/2 and to MpROXY1Δ14, where the 14 C‐terminal amino acids were removed, as well as to the 14 MpROXY1 C‐terminal amino acids alone (14AA). MpTGA and the MpTGA3xC variant, where all three cysteines were substituted by serines, were fused to the donor fluorophore mTURQUOISE2 (mTRQ2, Goedhart et al., 2012). Using the 2in1 system, the two fusion proteins were expressed from one plasmid, each under the control of the *CaMV 35S* promoter, which has been shown to enable the production of similar protein amounts in transient *N. benthamiana* transformation experiments (Grefen & Blatt, 2012; Hecker et al., 2015). In FRET experiments, upon interaction of the analyzed proteins, a nonradiant energy transfer from the donor to the acceptor fluorophore results in a decrease in the donor fluorescence lifetime if the two fluorophores are brought together to close proximity below 10 nm (Mueller, Galliardt, Schneider, Barisas, & Seidel, 2013). As a robust way to assess FRET, the excited state lifetime changes were recorded, which are independent of local chromophore concentrations and moderate photobleaching (Bhat, Lahaye, & Panstruga, 2006). Nuclear mTRQ2‐MpTGA proteins without an acceptor fluorophore showed a specific monoexponential fluorescence decay behavior resulting in a single lifetime of ~3.90 ns (Figure 3b,c). FRET leads to a biexponential decay behavior of mTRQ2 and thus results in two measurable lifetimes. The longer lifetime (τ_D_) represents fusion proteins that do not participate in the protein–protein interaction, while a reduced lifetime (τ_DA_) is due to an engagement in interactions (Becker, 2012). In the presence of the mVENUS‐MpROXY1 or mVENUS‐MpROXY2 interaction partners, two fluorescent lifetimes could be measured for the mTRQ2‐MpTGA. While the longer fluorescence lifetime was approximately 4 ns, the reduced lifetime was below 2 ns, proving the interaction of mTRQ2‐MpTGA with mVENUS‐MpROXY1/2 (Figure 3c). The deletion of the last 14 amino acids of the MpROXY1 C‐terminus revealed that no interaction occurred between MpTGA and MpROXY1Δ14 as only one mTRQ2 fluorescence lifetime could be determined (Figure 3b,c). Contrarily, the 14 amino acids from the MpROXY1 C‐terminus are sufficient to enable an interaction with MpTGA, as shown by the detection of a shortened lifetime in the mTRQ2‐MpTGA/mVENUS‐14AA FRET‐FLIM analysis (Figure 3c). These observations support the crucial function of the MpROXY1 C‐terminus for mediating interactions with MpTGA in vivo, which has also been shown for the ROXY1 C‐terminus in *A. thaliana* (Li et al., 2011). Mutagenesis of the three MpTGA cysteines to serines (MpTGA3xC) did not abrogate the protein interaction capability (Figure 3c). Therefore, the three MpTGA cysteine residues are not required for mediating an interaction with MpROXY1/2 proteins. Together, these analyses demonstrate that the nuclear interaction of CC‐type GRXs with TGA TFs has been conserved since their occurrence in basal land plants and that the interaction is mediated via the conserved ROXY C‐terminus.

### 3.4 Mp*ROXY1/2* and Mp*TGA* mRNA expression overlap

**Figure 4:**
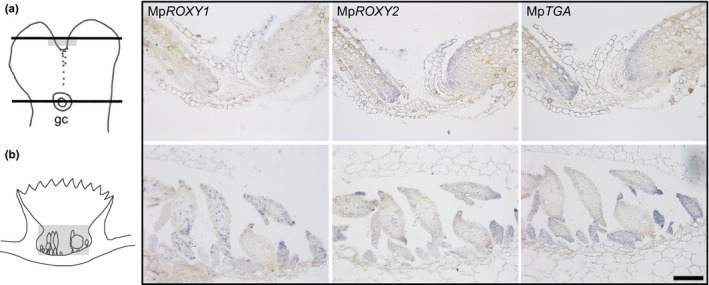
In situ mRNA hybridization analysis. Hybridization of Mp*ROXY1*, Mp*ROXY2,* and Mp*TGA* antisense probes on serial longitudinal sections revealed their overlapping expression in the meristematic zone of thallus tips (a) and in young gemmae that develop in gemma cups (b). Black lines in the scheme indicate the sectioning planes, and gray fields mark the depicted areas from the hybridized sections. gc, gemma cup. Bar = 100 μm

To further investigate the in vivo interaction potential of MpROXYs and MpTGA, we investigated whether their mRNA expression pattern overlap in different *M. polymorpha* tissues. As in other bryophytes, the gametophyte generation dominates the *M. polymorpha* life cycle and produces the main thalloid plant body. Thallus growth is achieved by cell divisions in the apical notch, a zone where the regular dichotomous branching of the expanding thallus is initiated. Asexual reproduction is realized via the formation of gemma cups on the dorsal thallus side, comprising clonal propagations units named gemmae (Shimamura, 2016; Figure 4). In situ RNA hybridizations were conducted on serial *M. polymorpha* thallus sections hybridized with antisense probes of the three respective transcripts to resolve their expression in thallus tissue and gemma cups (Figure 4). All genes are expressed in thallus tips, namely in the zone close to the apical notch (Figure 4a) as well as in young gemmae that develop in the gemma cups (Figure 4b). Whereas Mp*ROXY1* exhibits a punctuated expression pattern, Mp*ROXY2* mRNA is more broadly expressed in the tissue around the apical notch and in developing gemmae. The Mp*TGA* expression domain encompasses the regions of the Mp*ROXY1/2* expression, demonstrating that MpROXY1/2/MpTGA protein interactions can occur in planta in these investigated vegetative tissues and also in additional tissues, as indicated by the *M. polymorpha* transcriptome data (MapolyBase, Bowman et al., 2017).

### 3.5 MpTGA binds in a redox‐sensitive manner to the *as‐1*‐like element

**Figure 5:**
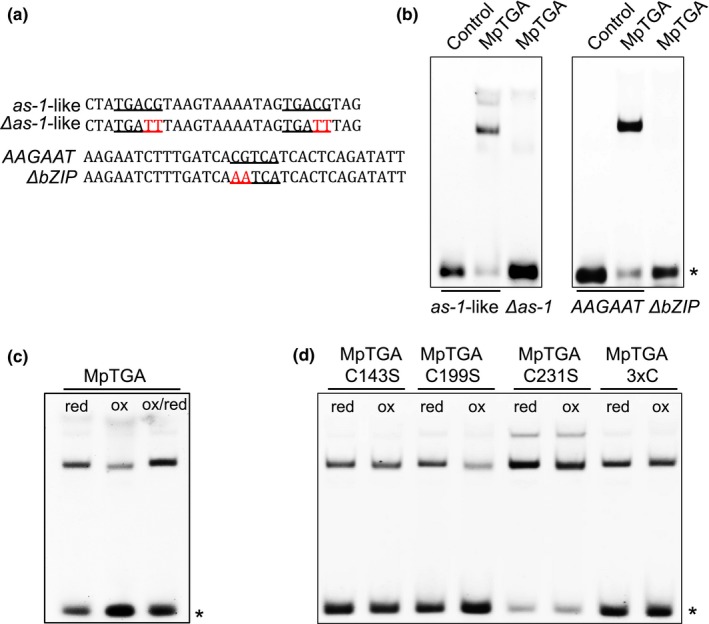
DNA binding EMSA analyses of MpTGA. (a) Sequences of the motifs used in the DNA binding studies. The *as‐1*‐like motif from the *A. thaliana PR1* promoter region contains two TGA core‐binding sites. The *AAGAAT* motif is present in the second intron of the *A. thaliana* floral homeotic regulator *AGAMOUS* and contains one central TGA core‐binding site. *Δas‐1*‐like and *ΔbZIP* motifs are the respective mutagenized versions, not mediating DNA binding. TGA core sequences are underlined, and mutagenized nucleotides in the *Δas‐1*‐like and *ΔbZIP* motifs are depicted in red. (B) EMSA analyses of the MpTGA interaction with the *as‐1*‐like, *Δas‐1*‐like (*Δas‐1)*,*AAGAAT,* and *ΔbZIP* motifs. Fluorescently labeled DNA probes were incubated with MpTGA protein or with a mock translation (control) under reducing conditions (0.9 mM DTT). (c) Analysis of redox‐sensitive MpTGA binding to the *as‐1*‐like motif. For comparison of reducing and oxidizing conditions, MpTGA protein was incubated prior to DNA binding with 0.9 mM DTT (red) and with 2 mM diamide (ox), respectively. Reversibility of the redox‐sensitive DNA binding was analyzed by adding 20 mM DTT after a 2 mM diamide treatment (ox/red). (d) To analyze the influence of the single MpTGAC143S, MpTGAC199S, MpTGAC231S, and triple MpTGA3xC variants, in vitro produced mutant protein was incubated either under reducing conditions (0.9 mM DTT; red) or under oxidizing conditions (2 mM diamide; ox). Asterisk marks the unbound DNA motifs

Recently, we demonstrated a redox‐modulated in vitro interaction of PAN with the *AAGAAT* motif from the second intron of the *A. thaliana* floral homeotic regulator *AGAMOUS* as well as with the *as‐1*‐like motif, a stress‐responsive element occurring in regulatory regions of diverse defense‐related genes such as the *A. thaliana PATHOGENESIS‐RELATED GENE1* (*PR1*) (Gutsche & Zachgo, 2016). The *as‐1*‐like motif contains two and the *AAGAAT* motif one central TGA TF core‐binding site. EMSA studies were conducted to investigate whether the *M. polymorpha* MpTGA protein also interacts with these motifs. MpTGA proteins were produced in vitro and incubated with fluorophore‐labeled *as‐1*‐like and *AAGAAT* oligonucleotides (Figure 5a). The mutagenized variants of these motifs, *Δas‐1*‐like and *ΔbZIP* (Figure 5a), contain nucleotide exchanges known to abrogate TGA DNA interactions and were included as negative controls (Gutsche & Zachgo, 2016; Maier et al., 2009). MpTGA proteins bind to the *as‐1*‐like and *AAGAAT* motif and specificities were confirmed with the mutagenized variants to which proteins could not bind (Figure 5b). The two shifted bands in the *as‐1*‐like interaction analysis indicate that MpTGA can likely bind to only one or to both of the two TGACG core motifs in this *cis*‐element, similar to what has been observed in *A. thaliana* TGA1 interaction studies (Lindermayr, Sell, Muller, Leister, & Durner, 2010). As the occurrence of the complete *AAGAAT* motif including the TGA core sequence is restricted to *AGAMOUS* homologs from eudicots (Gutsche & Zachgo, 2016), further EMSA studies were conducted with the *as‐1*‐like motif, which has a broader occurrence in diverse genes.

Next, we tested whether MpTGA exhibits like PAN a redox‐sensitive DNA binding. Redox EMSAs were conducted by incubating MpTGA with the reducing agent DTT (0.9 mM) or with the oxidant diamide (2 mM) prior to incubation with the *as‐1*‐like motif. Oxidizing conditions decreased the interaction of MpTGA with the DNA motif, an effect that could be reversed by adding a high DTT concentration (20 mM) to the oxidized reaction mix (Figure 5c). Redox‐sensitive DNA binding of PAN is mediated by the five Cys in the N‐terminal PAN extension, which exists only in PAN homologs of the Brassicaceae (Gutsche & Zachgo, 2016). The impact of the three Cys from MpTGA (Figure 1c) was analyzed by replacing all three Cys by Ser (MpTGA3xC), which abrogated the redox‐sensitive DNA binding (Figure 5d). Analysis of the individually mutagenized Cys143, Cys199, and Cys231 residues revealed that not a single cysteine but rather the joint activity of MpTGAC143 and MpTGAC231 mediates the redox sensitivity (Figure 5d).

### 3.6 MpROXY1/2 and MpTGA bind together to the *as‐1‐*like motif

**Figure 6:**
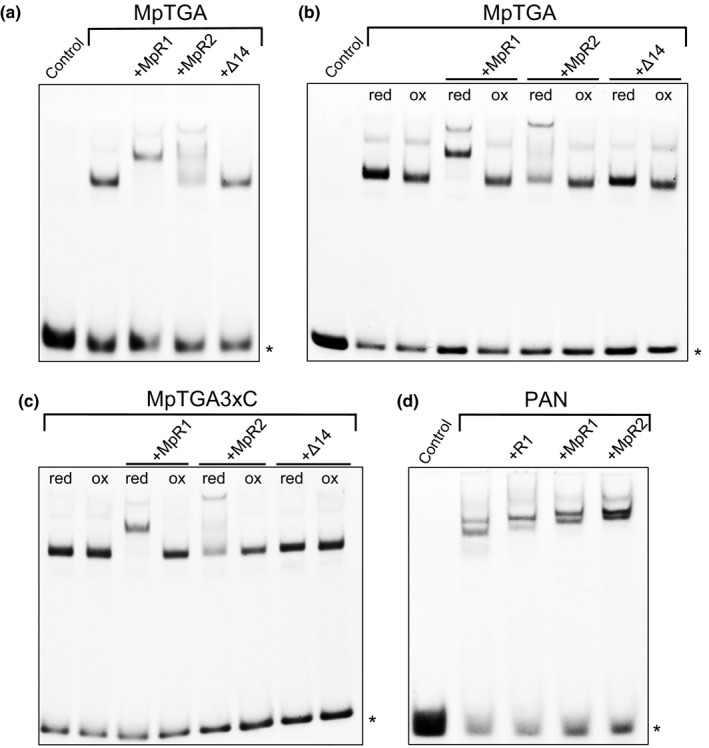
Binding studies of MpTGA and PAN together with MpROXY1/2. (a) EMSA analyses investigating the capacity of MpROXY1 (MpR1), MpROXY1 lacking the last 14 C‐terminal amino acids (Δ14), and MpROXY2 (MpR2) proteins to bind together with MpTGA to the *as‐1*‐like motif. (b) Redox EMSAs analyzing the impact of reducing (0.9 mM DTT) and oxidizing (2 mM diamide) conditions on the binding of wild‐type MpTGA and MpROXY1/2 proteins to the *as‐1*‐like motif. (c) DNA binding studies with the mutagenized MpTGA3xC protein and MpROXY1/2 under reducing (red) and oxidizing (ox) conditions. (d) Heterologous DNA binding studies investigating interactions of the *A. thaliana* TGA TF PAN with *A. thaliana* ROXY1 (R1), MpR1, and MpR2 protein. Asterisk marks the unbound *as‐1*‐like motif

Intrigued by the protein interaction studies showing that the ROXY1/TGA interaction evolved in basal land plants and that also *M. polymorpha* TGA proteins bind DNA in a redox‐sensitive manner, we investigated whether MpTGA bind in association with MpROXY1 and MpROXY2 to DNA. Both MpROXYs were expressed as MBP‐fusion proteins in *E. coli* and analyzed together with MpTGA proteins in DNA binding assays. Addition of MpROXY1/2 caused a supershift of the MpTGA*/as‐1*‐like bands, showing that MpROXY1/MpTGA as well as MpROXY2/MpTGA protein complexes interact with the *as‐1*‐like motif (Figure 6a). For MpROXY2, the supershift was not complete, indicating a stronger affinity of MpROXY1 to form DNA binding complexes with MpTGA. Neither MpROXY1/2 proteins nor MBP, used to facilitate protein expression and purification of the MpROXYs, did bind alone to the *as‐1*‐like motif (Fig. S5A).

Having demonstrated the importance of the MpROXY1 C‐terminus for the interaction with MpTGA (Figure 3b,c), EMSA studies were conducted using MBP‐MpROXY1Δ14 fusion proteins. Here, super‐shifted bands were no longer detectable as the lack of the C‐terminus abrogated the interaction of MpROXY1 with MpTGA (Figure 6a). Next, we analyzed the impact of redox‐modifying substances on the conjoined DNA binding of MpTGA and MpROXYs. The supershift, indicating complex formation of MpROXY1/2 and MpTGA with the *as‐1‐*like motif, occurred only under reducing conditions (Figure 6b). Furthermore, mutagenesis of the three MpTGA cysteines inhibits the redox sensitivity of the MpTGA DNA binding, but not the capability of the mutagenized MpTGA protein to bind together with MpROXY1/2 to the *as‐1*‐like element (Figure 6c). Together, our data show that the DNA binding of the MpTGA/MpROXY complex is redox‐dependent, occurring only under reducing conditions.

Given the conserved Y2H and BiFC interactions of *A. thaliana* and *M. polymorpha* CC‐type GRXs with TGA TFs (Figure 3, Fig. S3) as well as the heterologous *roxy1‐2* complementation experiments (Figure 2), we next investigated whether PAN/ROXY1 from *A. thaliana* and also heterologous PAN/MpROXY1/2 complexes can form and bind to the *as‐1*‐like element. As observed for MpTGA, also PAN proteins alone form two different complexes that interact with the element (Figure 6d). The addition of ROXY1, MpROXY1, and MpROXY2 proteins resulted in supershifts of the two bands, showing that homologous as well as heterologous complexes can interact with the *as‐1‐*like motif (Figure 6d), strengthening the highly conserved role of CC‐type GRXs in controlling TGA TF activities.

### 3.7 In vivo analysis of the MpROXY1 and MpROXY2 CC‐type motif

**Figure 7:**
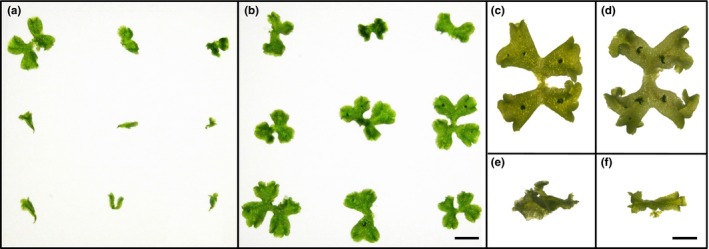
Analysis of the MpROXY1/2 CCxC motif. (a) Of nine randomly picked T1 overexpression plants, exemplarily shown for Mp*ROXY1‐OX* plants, only one showed a clear wild‐type‐like growth. (b) Replacement of the three cysteines in the CC‐type motifs of MpROXY1 and MpROXY2 abrogates the formation of the overexpression growth defects and is exemplarily shown for Mp*ROXY1‐OXAAMA* plants. (c–f) Comparison of four‐week‐old thalli from wild‐type (c), Mp*ROXY1‐OXAAMA* (d), Mp*ROXY1‐OX* (e), and Mp*ROXY2‐OX* (f) plants. Bar (a–b) = 1 cm and (c–f) = 5 mm

The activity of the *A. thaliana* CC‐type GRXs depends on their conserved motif (Xing et al., 2005; Zander et al., 2012), and we therefore investigated the impact of the CCxC motif for the Mp*ROXY1/2* activity in *M. polymorpha*. Mp*ROXY1* and Mp*ROXY2* CDS were expressed under the control of the Mp*EF1*α promoter shown to be suitable for ectopic expression studies (Althoff et al., 2014) and transgenic overexpression (OX) T1 plants were generated named Mp*ROXY1‐OX* and Mp*ROXY2‐OX*. The phenotypes of over one hundred randomly selected T1 plants were compared to wild‐type plants after 4 weeks. 70% of the Mp*ROXY1‐OX* plants (76/108 T1 plants, Figure 7a,e) and 61% of the Mp*ROXY2‐OX* plants (66/108 T1 plants, Figure 7f) revealed a severely reduced overall thallus growth. Additionally, thallus development was abnormal as no regular dichotomous branching occurred at the apex and no gemma cups with gemmae were formed. To test whether the Mp*ROXY1/2* overexpression activities depend on the presence of the cysteines in the CCxC motif, the three cysteines were mutagenized into alanines. In the transgenic T1 populations overexpressing Mp*ROXY1/2* with mutagenized CC motifs, the vast majority of the Mp*ROXY1‐OXAAMA* and Mp*ROXY2‐OXAAVA* plants resembled the wild‐type. Only 1.9% of the Mp*ROXY1‐OXAAMA* (2/107, Figure 7b) and 8.3% of the Mp*ROXY2‐OXAAVA* plants (9/108) showed growth defects similar to T1 lines overexpressing the wild‐type MpROXY1/2 proteins. These observations reveal the functional importance of this motif and its conserved cysteines. Furthermore, the growth defects in the overexpression lines indicate that a tightly regulated temporal and spatial CC‐type GRXs expression is crucial for the development of vegetative *M. polymorpha* tissues.

### 3.8 Nuclear ROXY1 colocalization analysis with active and inactive RNAPII

**Figure 8:**
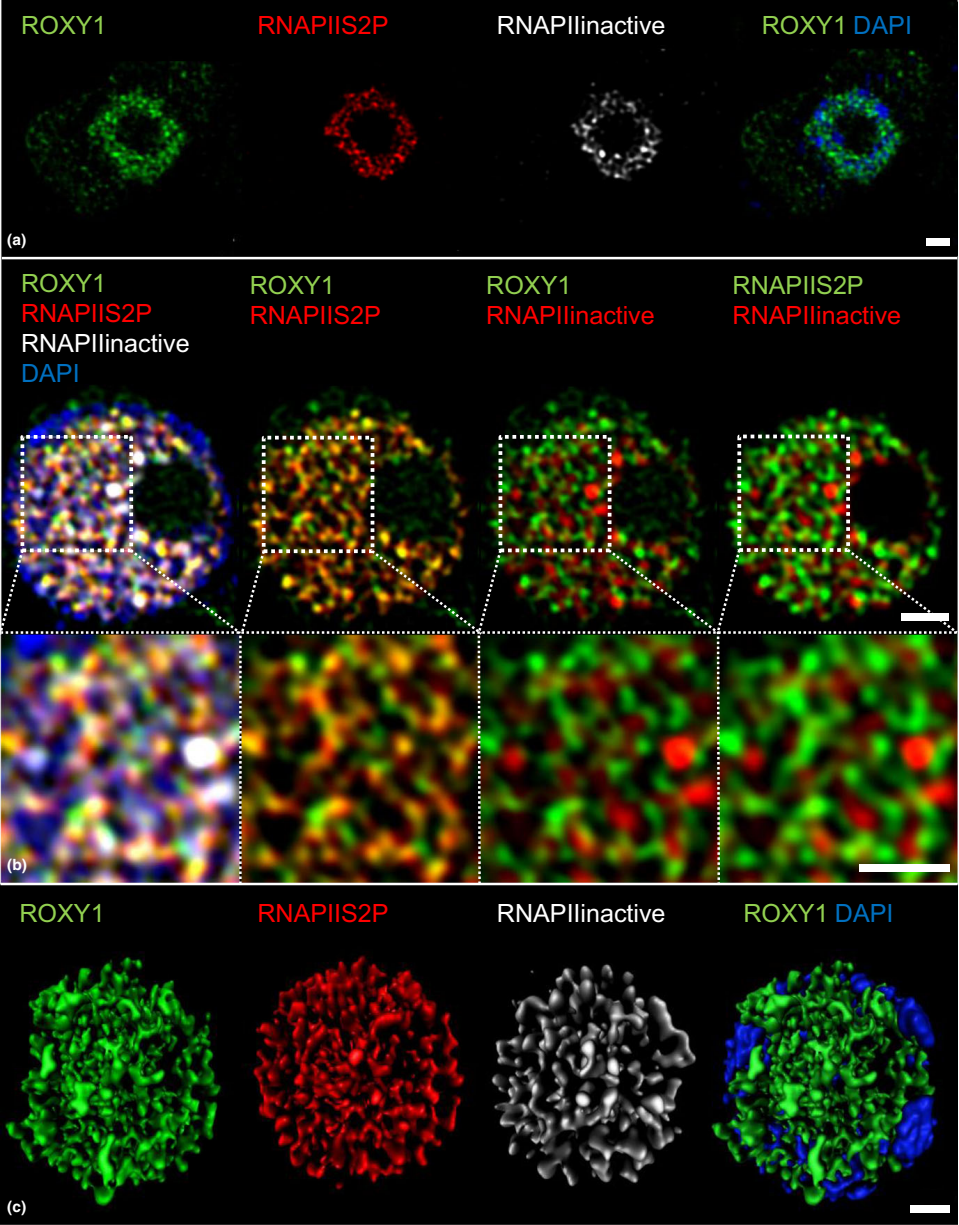
**Colocalization of ROXY1 and RNAPII in *A. thaliana* meristematic nuclei**. SIM colocalization analysis of ROXY1 with phosphorylated active RNAPII (RNAPIIS2P) and non‐phosphorylated inactive RNAPII proteins in meristematic *A. thaliana* root cells of *roxy1‐2* mutants complemented with GFP‐ROXY1 fusion proteins. Heterochromatin is visualized by DAPI. (A) The overview of the intracellular ROXY1 and RNAPII protein distribution shows a weak cytoplasmic and strong nuclear ROXY1 expression. (B) Nuclear colocalization analysis reveals a stronger association of ROXY1 with the active RNAPIIS2P than with the inactive form of RNAPII, which is indicated by a yellow color. Enlarged insets show the different degrees of colocalization and indicate that ROXY1 and RNAPII proteins form reticulate structures in the nucleoplasm, which is supported by the 3D reconstruction of nuclear SIM image stacks shown in (C). Bars = 1 μm

The highly conserved *ROXY* activities prompted us to investigate the subnuclear localization of ROXY1 in relation to transcription initiation processes in *A. thaliana*. Compared to widefield and deconvolution imaging, super‐resolution structured illumination microscopy (SIM) studies improve the classical Abbe/Rayleigh limit of ~250 nm up to an optical resolution of ~120 nm (Gustafsson, 2000; Gustafsson et al., 2008; Fig. S6A). SIM analyses were conducted with GFP‐ROXY1 fusion proteins that complemented the *A. thaliana roxy1‐2* mutant. GFP was N‐terminally fused to ROXY1 and expressed under the control of the 3.6‐kb *ROXY1* promoter, known to confer an endogenous *ROXY1* expression (Li et al., 2009; Xing et al., 2005). Four transgenic T1 lines with strong GFP expression in young flowers and a complemented, wild‐type‐like petal development were selected for further analyses. In addition to the *ROXY1* expression during flower development (Xing et al., 2005), *ROXY1* expression was also detected in the root meristem (Fig. S6B), revealing a strong nuclear and weaker cytoplasmic expression (Figure 8a). Recently, SIM has been applied to detect the subnuclear localization of the active and inactive form of the RNA polymerase II (RNAPII) in *A. thaliana* root meristem cells (Schubert, 2014; Schubert & Weisshart, 2015), which lack chloroplasts that exhibit a strong autofluorescence interfering with GFP analyses. This allowed investigating the colocalization of ROXY1 and RNAPII in root meristem cells at the subnuclear level. The carboxy‐terminal domain of the largest subunit of RNAPII is highly conserved and contains tandem heptapeptide repeats. These repeats provide recognition marks for post‐translational modifications to coordinate the recruitment of diverse nuclear proteins participating in transcription‐coupled events. Differential phosphorylation states of the repeats affect the transcriptional activity of RNAPII (Hajheidari, Koncz, & Eick, 2013). Antibodies specific for the active, elongating RNAPII, phosphorylated at serine 2 (RNAPIIS2P) and for the inactive, mostly nonphosphorylated RNAPII (RNAPIIinactive) were used for colocalization studies together with anti‐GFP serum to detect GFP‐ROXY1 fusion proteins.

The 3D‐SIM analysis of nuclei showed that ROXY1 proteins are localized in reticulate structures in the nucleoplasm and are absent from the nucleolus (Figure 8a,b). Counterstaining with DAPI visualized that ROXY1 is mainly localized in transcriptionally active euchromatin and not in the condensed and intensively DAPI‐stained heterochromatin (Figure 8c). Staining with RNAPIIS2P and RNAPIIinactive antibodies confirmed their known subnuclear localization (Schubert, 2014; Schubert and Weisshart. 2015, Figure 8a,c). After merging red RNAPIIS2P and green ROXY1 signals, strong yellow coloration indicates a large degree of ROXY1 colocalization with the active RNAPIIS2P form, which was not observed for the inactive RNAPII (Figure 8b). To compare the different colocalization degrees, the respective Pearson's correlation coefficients (PCC) were determined (Table S3). ROXY1 shows a higher association with RNAPIIS2P (PCC = 0.61 ± 0.1) than with RNAPII (PCC = 0.19 ± 0.07). RNAPII is known to form a reticulate network within the euchromatin of *A. thaliana* nuclei (Schubert, 2014) and the high degree of ROXY1 association with RNAPIIS2P argues for a similar subnuclear localization. A spatial surface rendering of whole ROXY1‐labeled nuclei was performed based on the SIM image stacks, which supports the formation of connected, reticulate structures (Figure 8c) resembling those described for RNAPII (Schubert, 2014). The strong colocalization with active RNAPIIS2P suggests that ROXY1 exerts a function in transcriptionally active networks.

## 4 DISCUSSION

### 4.1 The activity of ancestral CC‐type GRXs has been conserved during land plant evolution

The absence of CC‐type GRXs in chlorophytes and charophycean algae and their occurrence in two of the earliest divergent land plant lineages, the liverworts and mosses*,* argues that this novel land plant‐specific GRX class evolved in a common ancestor of the bryophytes. During land plant evolution, several independent gene duplication events contributed exclusively to a strong expansion of CC‐type GRXs (Ziemann et al., 2009). Therefore, genetic redundancies were generated providing the raw material for functional innovations by further diversification processes (Conant & Wolfe, 2008). In contrast to CC‐type GRXs, we identified TGA TFs in four species from charophycean algae. TGA TFs thus evolved before the CC‐type GRXs, likely in an ancestor of the streptophytes. Notably, diversification of land plants was not accompanied by a general, substantial evolution of novel TF families (Catarino et al., 2016). Similar to the majority of other plant TF families, the occurrence of TGA TFs also predates the colonization of land. Then, during land plant evolution, the expansion of the CC‐type GRXs was paralleled by an increase in the TGA TF family members. As ROXYs and TGA TFs exert regulatory functions in the same *A. thaliana* flower developmental and stress‐related processes (Gutsche, Thurow, Zachgo, & Gatz, 2015), we were intrigued to conduct comparative analyses with the respective orthologs from basal land plants, where they coexisted for the first time during land plant evolution.

MpROXY1 and MpROXY2 possess the typical CC‐type active site motifs, which are crucial for their activity as demonstrated by the Mp*ROXY1/2* overexpression analyses. Furthermore, all analyzed bryophyte CC‐type GRXs contain a C‐terminus with the LxxL/xL and ALWL motif shown to be required for the interaction with *A. thaliana* TGA TFs (Li et al., 2011; Zander et al., 2012). MpTGA contains the characteristic bZIP and two Q1/Q2 domains. Cys231 in the Q1 domain is at a position equivalent to Cys340 from PAN and Cys260 from TGA1, which were both shown to be required for the respective protein functions (Li et al., 2009; Lindermayr et al., 2010). Complementation studies of the *roxy1‐2* mutant with Mp*ROXY1/2* showed that the liverwort CC‐type GRXs can replace the *ROXY1* activity and govern normal petal formation in *A. thaliana*. Based on heterologous yeast and in planta BiFC interaction analyses, bryophyte ROXYs likely exert their activity in the *roxy1‐2* mutants via an interaction with TGA TFs. The capability to interact with TGA TFs thus seems to be an ancestral feature of CC‐type GRXs and depends on the presence of the last 14 C‐terminal MpROXY1/2 amino acids containing the LxxL/xL and ALWL motifs. These protein interactions are likely to occur in vivo, as the mRNA expression pattern of the three genes overlaps in the investigated *M. polymorpha* thallus and gemmae tissues. Our data demonstrate that the liverwort CC‐type GRXs exert biochemical activities that have been conserved for over 450 MY since the emergence of this novel GRX class in land plants.

### 4.2 MpTGA binds together with MpROXYs to the *as‐1*‐like motif

MpTGA binds redox sensitively to the regulatory *as‐1‐*like element. The *as‐1‐*like motif was first identified in the *CaMV 35S* promoter and represents one class of stress‐responsive elements widely used by defense‐related genes in angiosperms, namely the *PATHOGENESIS‐RELATED GENES* (Lam & Chua, 1989). For PAN, a redox‐sensitive DNA interaction is mediated by the combined activity of the five cysteines in the PAN‐specific N‐terminus (Gutsche & Zachgo, 2016). Analysis of the three MpTGA cysteines revealed that mainly the two cysteines Cys143 and Cys231 together contribute to this sensitivity. These findings propose that variable cysteine residues were recruited during the evolution of plant TGA TFs to regulate DNA binding in a redox‐sensitive manner. Other plant TFs such as the bHLH TF TCP15 are redox‐modulated in response to high light intensities (Viola, Camoirano, & Gonzalez, 2016), showing the importance of post‐translational TF redox modifications to adapt developmental regulatory networks in response to environmental changes.

Interestingly, redox EMSAs conducted with MpTGA and MpROXY1 or MpROXY2 demonstrate that these proteins bind under reducing conditions as complexes to the *as‐1*‐like element. The DNA complex formation was not affected by mutagenesis of the three MpTGA Cys, suggesting that its redox modulation is rather mediated by MpROXY1/2. Furthermore, the presence of the crucial MpROXY1 C‐terminal 14 amino acids is required for mediating an interaction with MpTGA and thereby also necessary for mediating the formation of a DNA‐bound complex under reducing conditions. ROXY19 from *A. thaliana* interacts with TGA2/5/6, but a redox‐sensitive TGA2/5/6 DNA binding has thus far not been reported as a mechanism to regulate TGA2/5/6 activities in stress responses (Herrera‐Vasquez et al., 2015; Zander et al., 2012). ROXY19 might rather affect the TGA2 activity via its interaction with TOPLESS, a transcriptional corepressor (Uhrig et al., 2017). However, as ROXY19 can complement the *roxy1* petal mutant phenotype (Li et al., 2009), it might thus also be capable to interact together with PAN at regulatory DNA elements, given it is expressed in a floral context. Together, these data suggest that the redox‐dependent DNA binding mechanism exhibited by MpROXY/MpTGA complexes represents a conserved, ancestral mode that contributes to the regulation of TGA protein activities.

### 4.3 *Arabidopsis thaliana* ROXY1 colocalizes with active RNAPII

To investigate the intracellular localization and subnuclear distribution of ROXYs as well as the correlation with active transcription processes, fluorescence SIM analyses were conducted. We employed the *roxy1‐2 A. thaliana* mutant complemented by GFP‐ROXY1 fusion proteins to analyze the ROXY1 expression in meristematic root cells. Investigation of the intracellular distribution showed a stronger ROXY1 localization in the nucleus compared to the cytoplasm. The communication between the cytosol and the nucleus is regulated by nuclear pore complexes, which are anchored in the nuclear envelope and mediate two different trafficking modes: passive diffusion of small molecules (<40–60 kDa) and transport receptor‐facilitated translocation of cargo molecules containing NLS (Fahrenkrog & Aebi, 2003). ROXY1 and other CC‐type GRXs lack a NLS and are rather small with masses of only ~12–14 kDa that should allow nuclear entry and exit via passive diffusion. However, the observed unequal intracellular ROXY1 distribution indicates that instead of free intracellular ROXY1 diffusion, these proteins seem to be retained in the nucleoplasm, which could be mediated via their association with nuclear TGA TFs.

The RNAPII is responsible for transcription of most eukaryotic protein‐coding genes, and we determined the colocalization of ROXY1 with its active and inactive forms. Recently, it was shown that both *A. thaliana* RNAPII variants are more evenly distributed within the euchromatin (Schubert, 2014; Schubert & Weisshart, 2015) than observed in mammalian nuclei, where RNAPII is organized in distinct domains, named transcription factories (Papantonis & Cook, 2013). Confirming these data, we also detected that RNAPII forms reticulate networks within the euchromatin. Super‐resolution SIM studies showed that ROXY1 colocalizes strongly with active RNAPIIS2P, whereas the colocalization of ROXY1 with inactive RNAPII is significantly weaker. RNAPII pausing has been shown to be a well‐established mechanism to control the timing, rate, and likely also the magnitude of transcriptional responses in yeast and humans (Liu, Kraus, & Bai, 2015). However, for *A. thaliana*, it has been shown that transcription might not be regulated by promoter‐pausing but rather at the level of transcription initiation (Hetzel, Duttke, Benner, & Chory, 2016). The emerging differences in plant transcription initiation regulation together with the observation that ROXY1 colocalizes with the active form of the RNAPII suggest that land plant‐specific CC‐type GRXs contributed to modulate transcription mechanisms, starting in the earliest land plants, the bryophytes.

## 5 CONCLUSIONS

Here, we show that regulatory *ROXY* functions and their interactions with TGA TFs as well as their redox‐dependent DNA binding together with TGA proteins were highly conserved during land plant evolution. Exclusively, the CC‐type GRX numbers increased during land plant evolution. In contrast to the CPYC and CGFS GRXs, which exhibit a more ubiquitous expression in vegetative and reproductive *A. thaliana* organs, the CC‐type *ROXYs* reveal more specialized expression patterns (Gutsche et al., 2015). Together with the data from this study, this supports the notion that *ROXY* activities were diversified rather by *cis*‐regulatory changes that established distinctive *ROXY* expression dynamics than by altering their biochemical activities. Variation in *ROXY* expression patterns enabled further sub‐ and neofunctionalization processes, which likely contributed to the recruitment of *ROXYs* into various developmental and stress‐related processes. The availability of molecular tools for *M. polymorpha* (Ishizaki, Nishihama, Yamato, & Kohchi, 2016; Kopischke, Schussler, Althoff, & Zachgo, 2017; Sugano et al., 2014) enables further functional analyses in one of the most basal land plants. This will help to advance our recently expanding knowledge on redox regulation of plant TF activities (Dietz, 2014) and can reveal how these processes contributed to adapt plants to novel challenges associated with a terrestrial lifestyle.

## AUTHOR CONTRIBUTIONS

N.G., M.H., L.M., M.O'D., A.B., V.S., and S.Z. planned the research and performed the data analysis. N.G., M.H., L.M., M.O'D., A.B., and A.L. conducted the experiments, and S.Z. supervised the experiments. N.G. and S.Z. wrote the manuscript with contributions of all authors.
