# Supplementary material for: Conserved redox‐dependent DNA binding of ROXY glutaredoxins with TGA transcription factors
Source: Plant Direct. 2017 Dec 14;1(6):e00030. doi: 10.1002/pld3.30 (PMC6508501; doi:10.1002/pld3.30)
Supplement: Supplementary file 1 [file PLD3-1-e00030-s001.pdf]

Supplementary Materials and Methods

Phylogenetic analyses of CC-type GRXs and TGA TFs in selected plant species

Protein sequences regarding CC-type GRXs and TGA TFs from the analyzed species (see Supplementary Table S1) were aligned using ClustalW from the MacVector program (version 12.0.2). The sequence alignments were manually trimmed to generate a TGA TF sequence alignment that includes the sequence regions from the bZIP domain until the Q2 domain. A group C bZIP TF from *A. thaliana* (At5g28770.2) and one from *M. polymorpha* (Mapoly0012s01702) were used as outgroups. For the CC-type GRXs, a 101 aa long alignment comprising the CC-type motif and the conserved C-terminal motifs was generated. As outgroups, the AtGRXC1 (At5g63030, CPYC) and AtGRXS17 (At4g04590, CGFS) were included. Phylogenetic analyses were done with 1,000 boostrap repetitions using the Neighbor Joining method in the uncorrected mode.

Yeast-2-Hybrid analysis

Full-length CDS of *PAN* (At1g68640) and *ROXY1* (At3g02000) from *A. thaliana* and Mp*TGA* (Mapoly0026s0039), Mp*ROXY1/2* and Mp*ROXY1* without the last 14 amino acids (Mp*ROXY1Δ14*) were amplified with flanking attB1 and attB2 sites and inserted into the pDONR™207 vector using the Gateway® technology (Invitrogen). Verified *ROXY* entry vectors were used together with the pDEST-GADT7 prey vector in LR reactions to generate destination vectors expressing MpROXY1/2 proteins N-terminally fused with GAL4-AD. The entry vectors were used for further cloning of Mp*TGA* into the bait vector pDEST-GBKT7 containing the GAL4-BD (Rossignol et al., 2007) and sequenced for verification. After cotransformation in the Y187 yeast strain, cells were plated on SD/-Trp -Leu selection medium (Clontech). Quantification of β-galactosidase activity was performed as described by Li et al. (2011).

In planta localization studies

Mp*ROXY1*, Mp*ROXY2* and Mp*TGA* pDONR™207 entry vectors were used together with the pGFP-N-BIN vector (originally generated by Ben Trevaskis, MPI-Golm, Potzdam, Germany) in LR reactions to generate destination vectors expressing MpROXY1, MpROXY2 and MpTGA proteins fused N-terminally to GFP. Agrobacterium-mediated transient transformation of *Nicotiana benthamiana* leaves was performed as described by Li et al. (2009). As a control the empty pGFP-N-BIN vector was transiently transformed into *N. benthamiana* leaves. Images were captured with a Zeiss 510 META NLO using a Plan-Apochromat 20x/0.8 objective.

For GFP-ROXY1 localization, roots of T3 *roxy1-2 A. thaliana* plants expressing GFP-ROXY1 under the control of the 3.6 kbp *ROXY1* promoter (see Materials and Methods) were used, which revealed a normal petal development. Confocal microscopy was performed using a Zeiss LSM 510 Meta NLO equipped with EC Plan-NeoFluar 40x/1.3 oil immersion objective. Plasma membranes were stained with FM4-64. GFP was excited via a 488 nm argon laser and its emission was detected using a 500 nm to 550 nm band-pass filter.

Supplementary Figure 1. Phylogenetic analysis of the CC-type GRXs in viridiplantae.

Phylogenetic analysis using the neighbor joining method with 1000 bootstrap replications were done based on a 101 aa long sequence alignment of CC-type GRXs including as outgroups the CPYC GRX AtGRXC1 (At5g63030) and CGFS GRX AtGRXS17 (At4g04590). The sequence alignment comprised characteristic amino acids such as the active site motif and the conserved C-terminal LxxL/xL and ALWL motifs. Sequences of *Marchantia polymorpha* (Mp), *Physcomitrella patens* (Pp), *Selaginella moellendorffii* (Smoe), *Picea abies* (Pab), *Pinus taeda* (Pta), *Brachypodium distachyon* (Bd), *Oryza sativa* (Os), *Mimulus guttatus* (Migut), *Populus trichocarpa* (Pt) *and Arabidopsis thaliana* (At) were used for the phylogenetic analysis. Nodes collapse under 50% and are labeled above 50%. The analyzed CC-type GRXs MpROXY1 and MpROXY2 from *M. polymorpha* are indicated in red. *A. thaliana* ROXYs are depicted in grey.

Supplementary Figure 2. Phylogenetic Neighbor-Joining tree of viridiplantae TGA TFs.

TGA TF protein sequences (group D bZIP TF, Jakoby et al., 2002) of *Klebsormidium flaccidum* (Kfl), *Nitella mirabilis* (GBST), *Coleochaete orbicularis* (GBSL), *Spirogyra pratensis* (GBSM), *Marchantia polymorpha* (Mp/Mapoly), *Physcomitrella patens* (Pp), *Selaginella moellendorffii* (Smoe), *Picea abies* (Pab), *Pinus taeda* (Pta), *Brachypodium distachyon* (Bd), *Oryza sativa* (Os), *Mimulus guttatus* (Migut), *Populus trichopoda* (Pt) and *Arabidopsis thaliana* (At) were aligned using ClustalW (MacVector, version 12.0.2). The alignment was manually trimmed before the bZIP domain and behind the Q2 region. At5G28770.2 and Mapoly0012s0172, two group C bZIP TFs (Jakoby et al., 2002), served as outgroups in a phylogenetic analysis using the neighbor joining method with 1,000 bootstrap replications. Nodes are labeled over 50% and collapse below 50%. Indicated are the five classes of the *A. thaliana* TGA TFs. MpTGA is marked in red and TGAs from *A. thaliana* are indicated in grey.

Supplementary Figure 3. ROXY/TGA interaction studies in yeast.

Y2H studies analyzed the interaction capacity of *M. polymorpha* and *A. thaliana* TGA TFs and ROXYs. The interaction strength for tested protein pairs was quantified by determining ß-galactosidase reporter gene expression (Miller Units, MU) as described previously (Li et al., 2011). As negative controls, cotransformation of empty bait and prey vectors (BD/AD) resulted in background levels of 0.20 to 1.41 MU. Similar values were obtained when the AD alone was coexpressed with the bait proteins PAN-BD and MpTGA-BD. Protein interactions were determined for MpROXY1-BD with MpTGA-AD (25.26 ± 3.5 MU) and MpROXY1 also interacts in the yeast system with PAN (13.46 ± 2.57 MU). Weaker interactions were detected for MpROXY2 with MpTGA (3.34 ± 0.60 MU) and PAN (2.33 ± 0.11 MU). Removal of the MpROXY1 C-terminal 14 amino acids (MpROXY1Δ14) precluded an interaction with MpTGA.

Supplementary Figure 4. In planta localization studies of MpTGA and MpROXY1/2.

(A) Reconstituted YFP fluorescence in BiFC interaction studies conducted in transiently transformed in tobacco leaves was not observed in cells expressing MpTGA, MpROXY1 and MpROXY2 combined with the respective complementary empty vector. (B) GFP-MpTGA proteins are localized in nuclei of *N. benthamiana* epidermal leaf cells. GFP-MpROXY1 and GFP-MpROXY2 proteins as well as GFP expressed alone were detected in the cytoplasm and nucleus. Bar = 50 µm.

Supplementary Figure 5. Control DNA-binding studies.

To further verify the formation of ROXY/TGA/DNA complexes, EMSA studies without TGA TF proteins were performed. (A) MBP-MpROXY1/2 as well as MBP protein alone were used in binding reactions with the *as-1*-like probe under reducing (red, 0.9 mM DTT) and oxidizing (ox, 2 mM diamide) conditions. (B) The effect of MBP alone on the PAN/DNA binding was investigated by analyzing

recombinantly produced MBP protein together with PAN. To test if ROXY1 (R1) from *A. thaliana* can bind alone to the *as-1*-like motif, recombinantly produced MBP-ROXY1 fusion protein was analyzed in EMSA studies. As a control, DNA probe was loaded alone on the native gel. Asterisk marks the unbound DNA motif.

Supplementary Figure 6. GFP-ROXY1 localization in Arabidopsis root meristem cells.

(A) Comparison of ROXY1, RNAPIIS2P, RNAPIIinactive and chromatin localization (DAPI) conducted by widefield microscopy, CLSM and SIM. Scale bar: 0.5 µm. (B) GFP-ROXY1 signals in the root meristem of a two-day-old complemented T3 *roxy1-2* seedling, expressing the GFP-ROXY1 fusion protein under the control of the endogenous *ROXY1* promoter. Plasma membranes were stained with FM4-64. Scale bar: 50 µm.

Supplementary Table S1. Accessions of GRXs and TGA TFs analyzed in Figure 1.

Supplementary Table S2. Oligonucleotide primers used in this study.

Supplementary Table 3. Degree of ROXY1 colocalization with RNAPII.

Colocalization of ROXY1 with active and inactive RNAPII was quantified by determining the Pearson‘s correlation coefficients, ranging from 1 to -1. 1 indicates a complete positive correlation and -1 a negative correlation, 0 stands for no correlation. Average values and standard deviations are based on the analysis of 22 nuclei after image processing using the Imaris 7.4 software.

Supplementary Materials and Methods

Phylogenetic analyses of CC-type GRXs and TGA TFs in selected plant species

Protein sequences regarding CC-type GRXs and TGA TFs from the analyzed species (see Supplementary Table S1) were aligned using ClustalW from the MacVector program (version 12.0.2). The sequence alignments were manually trimmed to generate a TGA TF sequence alignment that includes the sequence regions from the bZIP domain until the Q2 domain. A group C bZIP TF from *A. thaliana* (At5g28770.2) and one from *M. polymorpha* (Mapoly0012s01702) were used as outgroups. For the CC-type GRXs, a 101 aa long alignment comprising the CC-type motif and the conserved C-terminal motifs was generated. As outgroups, the AtGRXC1 (At5g63030, CPYC) and AtGRXS17 (At4g04590, CGFS) were included. Phylogenetic analyses were done with 1,000 boostrap repetitions using the Neighbor Joining method in the uncorrected mode.

Yeast-2-Hybrid analysis

Full-length CDS of *PAN* (At1g68640) and *ROXY1* (At3g02000) from *A. thaliana* and Mp*TGA* (Mapoly0026s0039), Mp*ROXY1/2* and Mp*ROXY1* without the last 14 amino acids (Mp*ROXY1Δ14*) were amplified with flanking attB1 and attB2 sites and inserted into the pDONR™207 vector using the Gateway® technology (Invitrogen). Verified *ROXY* entry vectors were used together with the pDEST-GADT7 prey vector in LR reactions to generate destination vectors expressing MpROXY1/2 proteins N-terminally fused with GAL4-AD. The entry vectors were used for further cloning of Mp*TGA* into the bait vector pDEST-GBKT7 containing the GAL4-BD (Rossignol et al., 2007) and sequenced for verification. After cotransformation in the Y187 yeast strain, cells were plated on SD/-Trp -Leu selection medium (Clontech). Quantification of β-galactosidase activity was performed as described by Li et al. (2011).

In planta localization studies

Mp*ROXY1*, Mp*ROXY2* and Mp*TGA* pDONR™207 entry vectors were used together with the pGFP-N-BIN vector (originally generated by Ben Trevaskis, MPI-Golm, Potzdam, Germany) in LR reactions to generate destination vectors expressing MpROXY1, MpROXY2 and MpTGA proteins fused N-terminally to

GFP. Agrobacterium-mediated transient transformation of *Nicotiana benthamiana* leaves was performed as described by Li et al. (2009). As a control the empty pGFP-N-BIN vector was transiently transformed into *N. benthamiana* leaves. Images were captured with a Zeiss 510 META NLO using a Plan-Apochromat 20x/0.8 objective.

For GFP-ROXY1 localization, roots of T3 *roxy1-2 A. thaliana* plants expressing GFP-ROXY1 under the control of the 3.6 kbp *ROXY1* promoter (see Materials and Methods) were used, which revealed a normal petal development. Confocal microscopy was performed using a Zeiss LSM 510 Meta NLO equipped with EC Plan-NeoFluar 40x/1.3 oil immersion objective. Plasma membranes were stained with FM4-64. GFP was excited via a 488 nm argon laser and its emission was detected using a 500 nm to 550 nm band-pass filter.

Supplementary Figure 1.

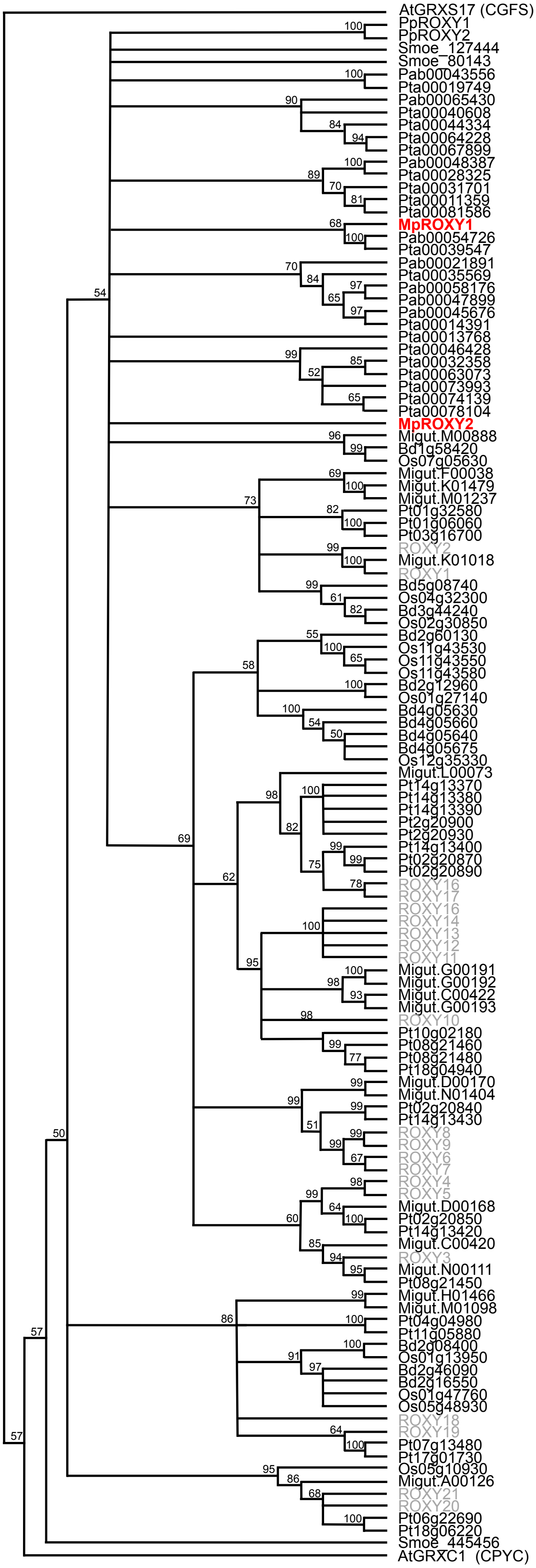

Supplementary Figure 1. Phylogenetic analysis of the CC-type GRXs in viridiplantae. Phylogenetic analysis using the neighbor joining method with 1000 bootstrap replications were done based on a 101 aa long sequence alignment of CC-type GRXs including as outgroups the CPYC GRX AtGRXC1 (At5g63030) and CGFS GRX AtGRXS17 (At4g04590). The sequence alignment comprised characteristic amino acids such as the active site motif and the conserved C-terminal LxxL/xL and ALWL motifs. Sequences of *Marchantia polymorpha* (Mp): *Physcomitrella patens* (Pp), *Selaginella moellendorffii* (Smoe), *Picea abies* (Pab), *Pinus taeda* (Pta), *Brachypodium distachyon* (Bd), *Oryza sativa* (Os), *Mimulus guttatus* (Migut), *Populus trichocarpa* (Pt) *and Arabidopsis thaliana* (At) were used for the phylogenetic analysis. Nodes collapse under 50% and are labeled above 50%. The analyzed CC-type GRXs MpROXY1 and MpROXY2 from *M. polymorpha* are indicated in red. *A. thaliana* ROXYs are depicted in grey.

Supplementary Figure 2.

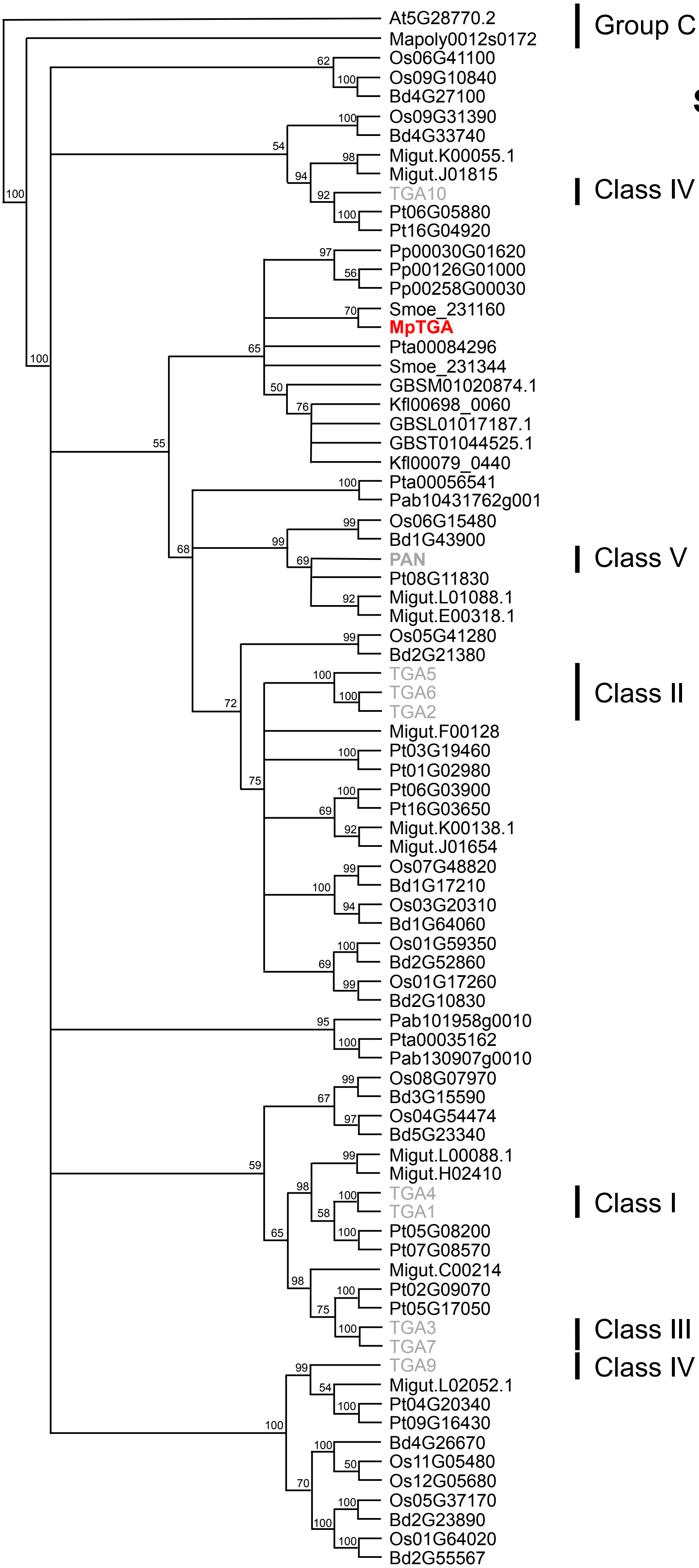

Supplementary Figure 2. Phylogenetic Neighbor-Joining tree of viridiplantae TGA TFs. TGA TF protein sequences (group D bZIP TF, Jakoby et al., 2002) of *Klebsormidium flaccidum* (Kfl), *Nitella mirabilis* (GBST), *Coleochaete orbicularis* (GBSL), *Spirogyra pratensis* (GBSM), *Marchantia polymorpha* (Mp/ Mapoly), *Physcomitrella patens* (Pp), *Selaginella moellendorffii* (Smoe), *Picea abies* (Pab), *Pinus taeda* (Pta), *Brachypodium distachyon* (Bd), *Oryza sativa* (Os), *Mimulus guttatus* (Migut), *Populus trichopoda* (Pt) and *Arabidopsis thaliana* (At) were aligned using ClustalW (MacVector, version 12.0.2). The alignment was manually trimmed before the bZIP domain and behind the Q2 region. At5G28770.2 and Mapoly0012s0172, two group C bZIP TFs (Jakoby et al., 2002), served as outgroups in a phylogenetic analysis using the neighbor joining method with 1.000 bootstrap replications. Nodes are labeled over 50% and collapse below 50%. Indicated are the five classes of the *A. thaliana* TGA TFs. MpTGA is marked in red and TGAs from *A. thaliana* are indicated in grey.

Supplementary Figure 3.

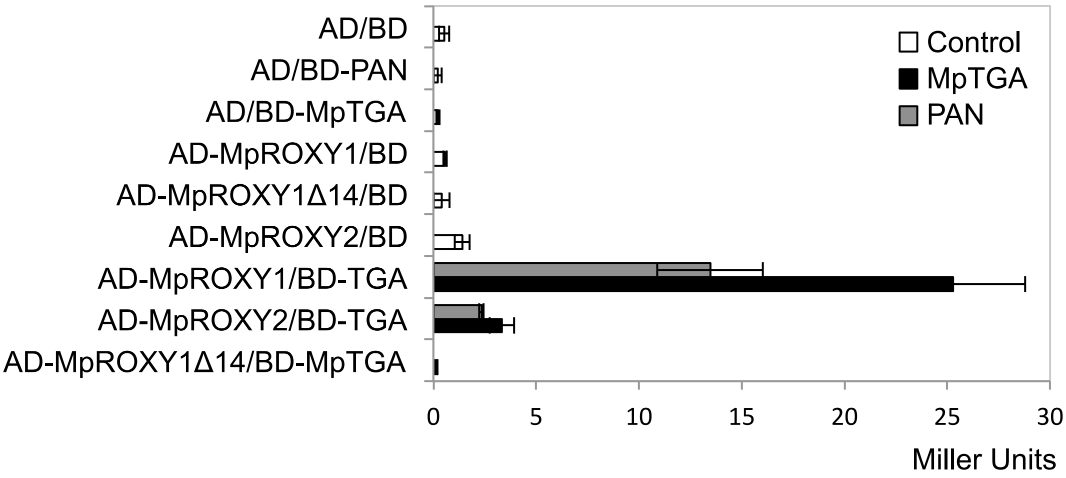

Supplementary Figure 3. ROXY/TGA interaction studies in yeast.
Y2H studies analyzed the interaction capacity of *M. polymorpha* and *A. thaliana* TGA TFs and ROXYs. The interaction strength for tested protein pairs was quantified by determining ß-galactosidase reporter gene expression (Miller Units, MU) as described previously (Li et al., 2011). As negative controls, cotransformation of empty bait and prey vectors (BD/AD) resulted in background levels of 0.20 to 1.41 MU. Similar values were obtained when the AD alone was coexpressed with the bait proteins PAN-BD and MpTGA-BD. Protein interactions were determined for MpROXY1-BD with MpTGA-AD (25.26 ± 3.5 MU) and MpROXY1 also interacts in the yeast system with PAN (13.46 ± 2.57 MU). Weaker interactions were detected for MpROXY2 with MpTGA (3.34 ± 0.60 MU) and PAN (2.33 ± 0.11 MU). Removal of the MpROXY1 C-terminal 14 amino acids (MpROXY1Δ14) precluded an interaction with MpTGA.

Supplementary Figure 4.

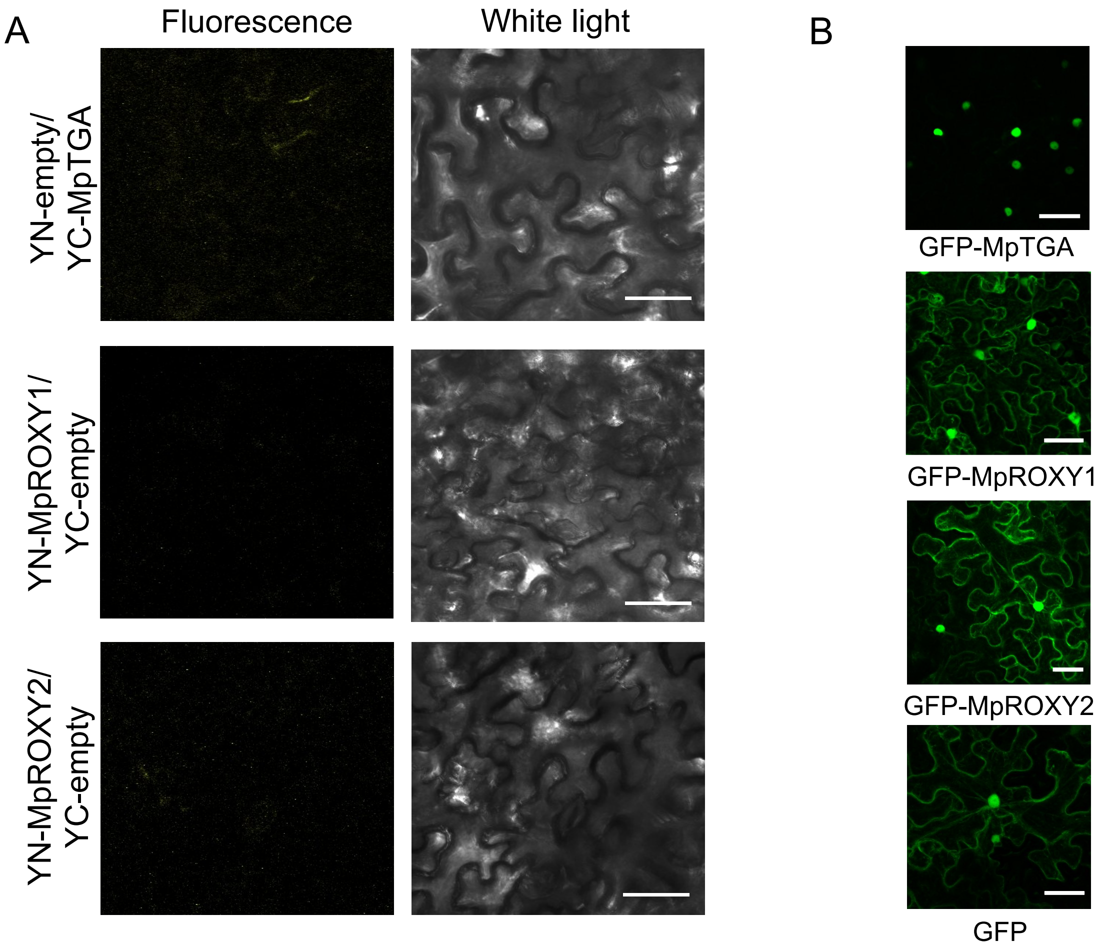

Supplementary Figure 4. In planta localization studies of MpTGA and MpROXY1/2.
(A) Reconstituted YFP fluorescence in BiFC interaction studies conducted in transiently transformed in tobacco leaves was not observed in cells expressing MpTGA, MpROXY1 and MpROXY2 combined with the respective complementary empty vector. (B) GFP-MpTGA proteins are localized in nuclei of *N. benthamiana* epidermal leaf cells. GFP-MpROXY1 and GFP-MpROXY2 proteins as well as GFP expressed alone were detected in the cytoplasm and nucleus. Bar = 50 µm.

Supplementary Figure 5.

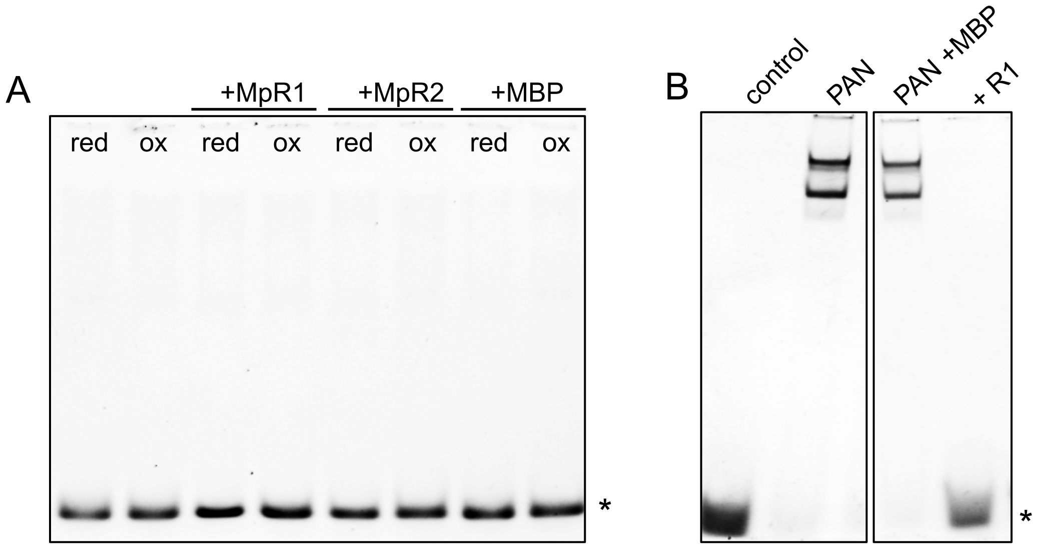

Supplementary Figure 5. Control DNA-binding studies.
To further verify the formation of ROXY/TGA/DNA complexes, EMSA studies without TGA TF proteins were performed. (A) MBP-MpROXY1/2 as well as MBP protein alone were used in binding reactions with the *as-1*-like probe under reducing (red, 0.9 mM DTT) and oxidizing (ox, 2 mM diamide) conditions. (B) The effect of MBP alone on the PAN/DNA binding was investigated by analyzing recombinantly produced MBP protein together with PAN. To test if ROXY1 (R1) from *A. thaliana* can bind alone to the *as-1*-like motif, recombinantly produced MBP-ROXY1 fusion protein was analyzed in EMSA studies. As a control, DNA probe was loaded alone on the native gel. Asterisk marks the unbound DNA motif.

Supplementary Figure 6.

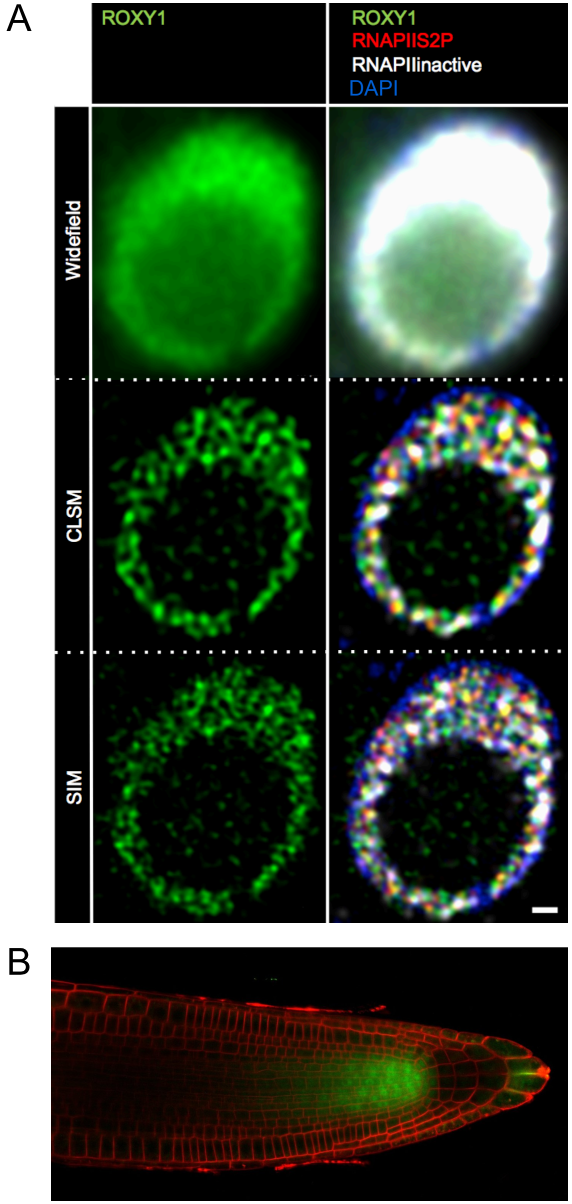

Supplementary Figure 6. GFP-ROXY1 localization in Arabidopsis root meristem cells.
(A) Comparison of ROXY1, RNAPIIS2P, RNAPIIinactive and chromatin localization (DAPI) conducted by widefield microscopy, CLSM and SIM. Scale bar: 0.5 µm. (B) GFP-ROXY1 signals in the root meristem of a two-day-old complemented T3 *roxy1-2* seedling, expressing the GFP-ROXY1 fusion protein under the control of the endogenous *ROXY1* promoter. Plasma membranes were stained with FM4-64. Scale bar: 50 µm.

**Supplementary Table 1**	**CGFS GRX**	**CPYC GRX**	**CC-type GRX**	**TGA TF**
Ostreococcus lucimarinus	Ol01g04470			
	Ol01g05000			
	Ol09g01950			
	Ol14g02690			
Chlamydomonas reinhardtii	Cre04.g232602.t1.1	Cre12.g550400.t1.2		
	Cre07.g325600.t1.1	Cre12.g513750.t1.1		
	Cre07.g325743.t1.1	Cre06.g278183.t1.1		
	Cre01.g047800.t1.1			
Klebsormidium flaccidum	kfl00024_0220	kfl00686_0010		Kfl00698_0060
	kfl00427_0110	kfl00330_0040		Kfl00079_0440
	kfl00144_0310	kfl00094_0060		
	kfl00061_430			
Nitella mirabilis	GBST01058060.1	GBST01045368.1		GBST01044525.1 (*translated sequence)
	GBST01075320.1	GBST01057808.1		
	GBST01042058.1	GBST01057807.1		
	GBST01042057.1	GBST01069313.1		
	GBST01094549.1	GBST01095980.1		
Coleochaete orbicularis	GBSL01050636.1	GBSL01050860.1		GBSL01017187.1 (* translated sequence)
	GBSL01050121.1	GBSL01008008.1		
	GBSL01007856.1	GBSL01008007.1		
	GBSL01050508.1	GBSL01014950.1		
		GBSL01030877.1		
Spirogyra pratensis	GBSM01023569.1	GBSM01023260.1		GBSM01020874.1 (*sligthly changed & translated)
	GBSM01022838.1	GBSM01023258.1		
	GBSM01023554.1	GBSM01002375.1		
	GBSM01023984.1	GBSM01002374.1		
		GBSM01002840.1		
Marchantia polymorpha	Mapoly0188s0015.1	Mapoly0182s0020.1	Mapoly0048s0012.1	Mapoly0026s0039.1
	Mapoly0001s0103.1	Mapoly0078s0046.1	Mapoly0059s0028.1	
	Mapoly0006s0309.1	Mapoly0078s0047.1		
	Mapoly0037s0022.1	Mapoly0004s0084.1		
Physcomitrella patens	Pp00001g02010	Pp00042g00200	Pp00125g00550	Pp00030g01620
	Pp00006g02240	Pp00135g06590	Pp00063g00030	Pp00126g01000
	Pp00051g00830	Pp00131g00110		Pp00258g00030
	Pp00079g00740	Pp00185g00020		
	Pp00126g00380	Pp00321g00030		
	Pp00212g00550			
	Pp00403g00130			
	Phpat008g070400.1			
Selaginella moellendorffii	Smoe_57444	Smoe_229902	Smoe_80143	Smoe_231160

Selaginella moellendorffii	**CGFS GRX**	**CPYC GRX**	**CC-type GRX**	**TGA TF**
	Smoe_123396	Smoe_89111	Smoe_127444	Smoe_231344
	Smoe_102863	Smoe_19369	Smoe_445456	
	Smoe_85254	Smoe_174566		
	Smoe_88036			
Picea abies	MA_10436042g0030	MA_6584913g0010	Pab00065430	Pab10431762g0010
	MA_85544g0010	Pab00006062	Pab00054726	Pab130907g0010
	MA_794114		Pab00058176	Pab101958g0010
	MA_659551g0010		Pab00047899	
			Pab00021891	
			Pab00048387	
			Pab00045676	
			Pab00043556	
Pinus taeda	Pta00006313	Pta00026669	Pta00081586	Pta00035162
	Pta00006747	Pta00042058	Pta00078104	Pta00056541
	Pta00042093	Pta00031728	Pta00074139	Pta00084296
	Pta00043642	Pta00067654	Pta00073993	
	Pta00048509		Pta00067899	
			Pta00013768	
			Pta00011359	
			Pta00014391	
			Pta00019749	
			Pta00028325	
			Pta00031701	
			Pta00032358	
			Pta00035569	
			Pta00039547	
			Pta00040608	
			Pta00044334	
			Pta00046428	
			Pta00063073	
			Pta00064228	
Brachypodium distachyon	Bd1g01570	Bd1g31450	Bd1g58420	Bd1g43900
	Bd1g08020	Bd3g12720	Bd2g08400	Bd1g64060
	Bd2g04637	Bd3g50172	Bd2g12960	Bd2g10830
	Bd3g30080	Bd5g15220	Bd2g16550	Bd2g21380
	Bd4g45030		Bd2g46090	Bd2g23890
			Bd2g60130	Bd2g52860
			Bd3g44240	Bd2g55567
			Bd4g05630	Bd3g15590
			Bd4g05640	Bd4g26670
			Bd4g05660	Bd4g27100

Brachypodium distachyon	**CGFS GRX**	**CPYC GRX**	**CC-type GRX**	**TGA TF**
			Bd4g05675	Bd4g33740
			Bd5g08740	Bd5g23340
				Bd1g17210
Oryza sativa	Os12g07650	Os02g40500	Os01g13950	Os01g59350
	Os10g35720	Os02g43180	Os01g27140	Os01g64020
	Os01g34620	Os04g42930	Os01g47760	Os03g20310
	Os01g07950	Os06g44910	Os02g30850	Os04g54474
	Os03g63420	Os08g45140	Os04g32300	Os05g37170
			Os05g10930	Os05g41280
			Os05g48930	Os06g15480
			Os07g05630	Os06g41100
			Os11g43530	Os07g48820
			Os11g43550	Os08g07970
			Os11g43580	Os09g10840
			Os12g35330	Os09g31390
				Os11g05480
				Os12g05680
				Os01g17260
Mimulus guttatus	Migut.M01149	Migut.H00093	Migut.K01018	Migut.F00128
	Migut.B01277	Migut.O00117	Migut.F00038	Migut.J01654
	Migut.F00004	Migut.K00226	Migut.M01237	Migut.K00138
	Migut.L02023	Migut.N02241	Migut.K01479	Migut.L02052
		Migut.D00496	Migut.C00420	Migut.L01088
		Migut.H02493	Migut.M00888	Migut.K00055
			Migut.N00111	Migut.E00318
			Migut.D00170	Migut.H02410
			Migut.G00191	Migut.J01815
			Migut.G00192	Migut.C00214
			Migut.G00193	Migut.L00088
			Migut.N01404	
			Migut.D00168	
			Migut.C00422	
			Migut.H01466	
			Migut.M01098	
			Migut.L00073	
			Migut.A00126	
Populus trichopoda	Pt03g06070	Pt01g34770	Pt01g06060	Pt01g02980
	Pt16g11920	Pt07g01730	Pt01g32580	Pt02g09070
	Pt04g04220	Pt12g08280	Pt02g20840	Pt03g19460
	Pt11g05140	Pt15g07890	Pt02g20850	Pt04g20340
	Pt14g14120	Pt18g13340	Pt02g20870	Pt05g08200

Populus trichopoda	**CGFS GRX**	**CPYC GRX**	**CC-type GRX**	**TGA TF**
		Pt02g25410	Pt02g20890	Pt05g17050
			Pt02g20900	Pt06g03900
			Pt02g20930	Pt06g05880
			Pt03g16700	Pt07g08570
			Pt04g04980	Pt08g11830
			Pt06g22690	Pt09g16430
			Pt07g13480	Pt16g03650
			Pt08g21450	Pt16g04920
			Pt08g21460	
			Pt08g21480	
			Pt10g02180	
			Pt11g05880	
			Pt14g13370	
			Pt14g13380	
			Pt14g13390	
			Pt14g13400	
			Pt14g13420	
			Pt14g13430	
			Pt17g01730	
			Pt18g04940	
			Pt18g06220	
Arabidopsis thaliana	At2g28270	At1g77370	At4g33040	At1g08320
	At3g15660	At2g20270	At5g11930	At1g22070
	At3g54900	At4g28730	At1g03850	At1g68640
	At4g04590	At5g63030	At1g28480	At1g77920
		At5g40370	At1g06830	At3g12250
		At5g20500	At2g30540	At5g06839
			At3g62960	At5g06950
			At2g47880	At5g06960
			At2g47870	At5g10030
			At3g62950	At5g65210
			At3g21460	
			At5g14070	
			At3g02000	
			At5g18600	
			At4g15700	
			At4g15690	
			At4g15680	
			At4g15670	
			At4g15660	
			At1g03020	

Arabidopsis thaliana	**CGFS GRX**	**CPYC GRX**	**CC-type GRX**	**TGA TF**
			At3g62930	

Supplementatary Table 2

Primer list

Restriction sites, *att*B sites and T7 polymerase binding-sites are indicated in bold.

roxy1-2 compl.			
	ROXY1(GFP)F	AAA**TCTAGA**ATGGGTAAAGGAGAAGAACTTTTCACTGG	*Xba*I
	ROXY1(GFP)R	AAA**TCTAGA**GCCACCCCCTCCTTTGTATAGTTCATCCATGCC	*Xba*I
	MpROXY1F	AA**TCTAGA**ATGCAGACTCAGACACAGTC	*Xba*I
	MpROXY1R	AA**TCTAGA**TTCAAAGCCACAGCGCTCC	*Xba*I
	MpROXY2F	AA**TCTAGA**TCTATAGCCAGAGAGCGCC	*Xba*I
	MpROXY2R	AA**TCTAGA**ATGCAGAGCCCGCACCCGTTT	*Xba*I
Y2H/BiFC	MpTGA F	**GGGGACAAGTTTGTACAAAAAAGCAGGCTTA**ATGGCTGATAATAGCCCCCGTAC	*att*B
	MpTGA R	**GGGGACCACTTTGTACAAGAAAGCTGGGTC**TCAATCTCTAGGACGAGCAGACC	*att*B
	PAN F	**GGGGACAAGTTTGTACAAAAAAGCAGGCTTC**ATGCAGAGCAGCTTCAAAACC	*attB*
	PAN R	**GGGGACCACTTTGTACAAGAAAGCTGGGTG**TTAGTCTCTAGGTCTGGCTAACCATA	*attB*
Y2H	MpROXY1Δ14R	**GGGGACCACTTTGTACAAGAAAGCTGGGTC**TCACCCGCTGATGTGCGCCGCCATC	*att*B
FRET-FLIM	MpTGAF	**GGGGACAACTTTGTATAATAAAGTTGTA**ATGGCTGATAATAGCCCCCGTAC	*att*B
	MpTGAR	**GGGGACCACTTTGTACAAGAAAGCTGGGT**TCAATCTCTAGGACGAGCAGACC	*att*B
	MpROXY1F	**GGGGACAAGTTTGTACAAAAAAGCAGGCTTA**ATGCAGACTCAGACACAGTCGC	*att*B
	MpROXY1R	**GGGGACAACTTTGTATAGAAAAGTTGGGTG**TCAAAGCCACAGCGCTCCGGC	*att*B
	MpROXY2F	**GGGGACAAGTTTGTACAAAAAAGCAGGCTTA**ATGCAGAGCCCGCACCCGCAATC	attB
	MpROXY2R	**GGGGACAACTTTGTATAGAAAAGTTGGGTG**CTATAGCCAGAGAGCGCCGGC	*att*B
	MpROXY1 Δ14R	**GGGGACAACTTTGTATAGAAAAGTTGGGTG**TCACCCGCTGATGTGCGCCGCCATC	*att*B
pMH-mCHERRY	Non-codingF	CCC**AAGCTT**GGGCGCGCCTTAGGTGGCGGTACTTGGGTC	*Hind*III
	Non-codingR	GGC**GAGCTC**GCCTTAATTAAGGATGTTACGCAGCAGCAACGATG	*Sac*I
	proMpEF1αF	CCC**AAGCTT**GGGCAAATGAGTCACACACATTGTTG	*Hind*III
	proMpEF1αR	AA**GGCGCGCC**CCAACCTTTCTGCAGGCACATC	*Asc*I
	mCHERRYF	TT**GGCGCGCC**AAATGGTGAGCAAGGGCGAGGAG	*Asc*I
	mCHERRYR	GCTTTTTTGTACAAACTTGTGATCTTGTACAGCTCGTCCATGCC	
	GATEWAYF	GGCATGGACGAGCTGTACAAGATCACAAGTTTGTACAAAAAAG	

		C	
	GATEWAYR	CC**TTAATTAA**GGCACCACTTTGTACAAGAAAGC	*Pac*I
OX in Mp/Y2H/BiFC	MpROXY1F	**GGGGACAAGTTTGTACAAAAAAGCAGGCTTA**ATGCAGACTCAGACACAGTCGC	*att*B
	MpROXY1R	**GGGGACCACTTTGTACAAGAAAGCTGGGTC**TCAAAGCCACAGCGCTCCGGCTTC	*att*B
	MpROXY2F	**GGGGACAAGTTTGTACAAAAAAGCAGGCTTA**ATGCAGAGCCCGCACCCGCAATC	*att*B
	MpROXY2R	**GGGGACCACTTTGTACAAGAAAGCTGGGTC**CTATAGCCAGAGAGCGCCGGC	*att*B
EMSAs			
	as-1-likeF	CTACGTCACTATTTTACTTACGTCATAG	
	as-1-likeR	CTATGACGTAAGTAAAATAGTGACGTAG	
	Δas-1-likeF	CT**AA**ATCACTATTTTACTT**AA**ATCATAG	
	Δas-1-likeR	CTATGA**TT**TAAGTAAAATAGTGA**TT**TAG	
	AAGAATF	AAGAATCTTTGATCACGTCATCACTCAGATATT	
	AAGAATR	AATATCTGAGTGATGACGTGATCAAAGATTCTT	
	ΔbZIPF	AAGAATCTTTGATCA**AA**TCATCACTCAGATATT	
	ΔbZIPR	AATATCTGAGTGATGATTTGATCAAAGATTCTT	
	MpTGAF	GC**GGTACC**ATGGCTGATAATAGCC	*Kpn*I
	MpTGAR	GC**TCTAGA**TCAATCTCTAGGACGAGC	*Xba*I
	MpTGAC143SF	GATCCGTCAAATG**TCC**GACCTTCGTGCCGC	
	MpTGAC143SR	CAATGCGGCACGAAGGTC**GGA**CATTTGACG	
	MpTGAC199SF	AGACACCAGCTGAGCGG**TCT**TTCATGTGGA	
	MpTGAC199SR	TCCTCCCATCCACATGAA**AGA**CCGCTC	
	MpTGAC231SF	AGTTGCTTGGTATC**TCC**AATCTTCAACAGTCG	
	MpTGAC231SR	GCGACGACTGTTGAAGATT**GGA**GATACCAA	
pMAL	MpROXY1F	TGCTCG**GAAGGATTTC**GCAGACTCA	*Xmn*I
	MpROXY1R	TACTCA**GGATCC**TCAAAGCCACAGC	*Bam*HI
	MpR1AAMAF	TTGAGTTCT**GCAGCT**ATG**GCA**CATGTGGTGAAG	
	MpR1AAMAR	GCGCTTCACCACATGTGCCATAGCTGCAGAACT	
	MpROXY1Δ14R	GC**GGATCC**TCACCCGCTGATGTG	*Bam*HI
	MpROXY2F	TGCTCG**GAAGGATTTC**GCAGAGCCCGC	*Xmn*I
	MpROXY2R	TACTCA**GGATCC**CTATAGCCAGAGAGC	*Bam*HI
	MpROXY2AAVAF	AGCTCG**GCCGCC**GTG**GCC**CATGTGGTGAAGCGGCT	
	MpROXY2AAVAR	TTCACCACATGGGCCACGGCGGCCGAGCTGATGCT	
In situ hybrid.			
	MpROXY1F	TCTAGTCGACATCAGTCGCATTGG	
	MpROXY1R	TGCG**TAATACGACTCACTATAGGG**ATGTACCACTCTGC	T7
	MpROXY2F	TCTAAAGCTTATCCAGAGTGTTCG	
	MpROXY2R	TGCG**TAATACGACTCACTATAGGG**GTTCTACTGATCC	T7
	MpTGAF	TCTAGTCGACAGATCACACTGG	
	MpTGAR	TGCG**TAATACGACTCACTATAGGG**AGCTGATCAAGC	T7

Supplementary Table 3.

ROXY1<br>DAPI	RNAIIS2P<br>DAPI	RNAPIIinactive<br>DAPI	ROXY1<br>RNAIIS2P	ROXY1<br>RNAPIIinactive	RNAIIS2P<br>RNAPIIinactive
- 0.02<br>(± 0.07)	- 0.01<br>(± 0.02)	- 0.04<br>(± 0.03)	0.61<br>(± 0.1)	0.19<br>(± 0.07)	0.27<br>(± 0.08)

Supplementary Table 3. Degree of ROXY1 colocalization with RNAPII.
Colocalization of ROXY1 with active and inactive RNAPII was quantified by determining the Pearson‘s correlation coefficients, ranging from 1 to -1. 1 indicates a complete positive correlation and -1 a negative correlation, 0 stands for no correlation. Average values and standard deviations are based on the analysis of 22 nuclei after image processing using the Imaris 7.4 software.
